# Prenatal exposure to green space and mental health in early adolescence: findings from the TRAILS study

**DOI:** 10.1093/aje/kwae373

**Published:** 2024-09-24

**Authors:** Yi Zeng, Gonneke W J M Stevens, Tomáš Paus, Marco Helbich

**Affiliations:** Department of Human Geography and Spatial Planning, Faculty of Geosciences, Utrecht University, Utrecht, the Netherlands; Department of Interdisciplinary Social Science, Faculty of Social and Behavioural Sciences, Utrecht University, Utrecht, the Netherlands; Centre Hospitalier Universitaire Sainte-Justine, University of Montreal, Montreal, Quebec, Canada; Departments of Psychiatry and Neuroscience, Faculty of Medicine, University of Montreal, Montreal, Quebec, Canada; ECOGENE-21, Chicoutimi, Quebec, Canada; Department of Human Geography and Spatial Planning, Faculty of Geosciences, Utrecht University, Utrecht, the Netherlands

**Keywords:** green space, mental health, prenatal exposure, adolescence, life course, externalizing problems, internalizing problems, substance use

## Abstract

Little is known about whether green space exposure prenatally contributes to mental health later in life. Using data from a Dutch cohort (Tracking Adolescents’ Individual Lives Survey; *n* = 1476), we assessed associations between green space exposure prenatally (1989-1991) and 4 mental health outcomes: externalizing problems, internalizing problems, tobacco use, and alcohol use, self-reported at age 11 years (2001-2002), and we assessed mediation of gestational age and birth weight on these associations. In a structural equation model, adolescents with 1 SD unit more green space exposure prenatally had 0.119 SD (95% CI, 0.028-0.210) more externalizing problems in early adolescence. There are 2 potential explanations for this unexpected positive association. First, controlling for urbanicity attenuated this association to become non-significant, but the degree of attenuation was minor (0.096; 95% CI, −0.003 to 0.195). Second, this unexpected association might be a consequence of changes in green space exposure in the intervening years, namely childhood (from birth to early adolescence), indicating that individuals with increased green space exposure over childhood had fewer externalizing problems in early adolescence. For the prenatal green space–externalizing problems association, we did not observe mediation by gestational age or birth weight. Overall, these findings suggest no beneficial role of prenatal exposure to green space on adolescent mental health. Instead, increased green space exposure in childhood may lead to fewer externalizing problems in early adolescence.

## Introduction

Adolescence is a critical phase for mental development: greater than 50% of long-term mental health problems start in this life stage.^1^ There is a growing interest in understanding area-level determinants of adolescent mental health,^2^ largely driven by the recognition of the modifiable nature of these factors, which are crucial for location-specific health interventions.^3^ Exposure to green space has been suggested to be supportive of mental health, potentially through pathways of attention restoration, stress reduction, promotion of physical activities, and mitigation of environmental stressors.^4^^-^^6^

Prior studies, often cross-sectional, largely assessed if adolescents’ mental health is associated with the available green space in the neighborhood where the adolescents currently reside.^2^ Little is known, however, about whether mental health in adolescence is associated with exposure to green space earlier in life. A few longitudinal studies traced green space exposure back to childhood, reporting better mental health in adolescents with more exposure in childhood^7^^-^^9^; others found null or mixed associations.^10^^,^^11^

The beneficial role of green space exposure may date back even further, to the prenatal period, a potentially sensitive period to external exposures due to rapid development of brain structures and cognitive functioning in fetuses.^12^^-^^14^ In turn, early brain development is related to mental illness.^15^ Studies of this potential long-term green space–mental health linkage, however, are scarce.^16^^-^^18^ Pagalan et al.^16^ and Chen et al.^18^ found a slightly lower risk of autism spectrum disorders in children exposed to more green space prenatally. In contrast, Maitre et al.^17^ reported heightened externalizing problems in children with more prenatal exposure to green space. It has been unclear whether the association between prenatal exposure to green space and later-life mental health varies across types of mental health outcomes and whether such an association lasts into adolescence.

If present, the underlying mechanisms responsible for the association between prenatal exposure to green space and mental health later in life remain unclear. Birth outcomes might explain this association. Although previous findings are not entirely consistent, there are clear indications that prenatal exposure to green space was protective against adverse birth outcomes, including preterm birth (gestational age < 37 weeks) and low birth weight (<2500 g),^19^^,^^20^ potentially through influencing fetal growth in utero and placental epigenetic mechanisms.^21^^-^^24^ These birth outcomes were suggested to be associated with restricted growth of the cerebral cortex,^25^^,^^26^ which, in turn, might be related to higher risks of developing mental health problems.^27^ Even so, there is still no direct evidence showing whether the potential effects of prenatal exposure to green space on birth outcomes could translate into mental health development later in life.

To fill these knowledge gaps, first we investigated the association between prenatal exposure to green space and mental health in early adolescence. We focused on 3 dimensions of mental health problems: externalizing problems, internalizing problems, and substance use (tobacco and alcohol). Second, we tested whether gestational age and birth weight mediated the associations between prenatal exposure to green space and mental health problems in early adolescence. We hypothesized that more prenatal exposure to green space is associated with fewer externalizing problems and internalizing problems, and less tobacco use and alcohol use in early adolescence, and these associations are mediated by higher gestational age and birth weight.

## Methods

### Study population

We used longitudinal data from the Tracking Adolescents’ Individual Lives Survey (TRAILS), a prospective cohort study conducted in the northern Netherlands. TRAILS started in 2001 and requested participation of all primary schools in the provinces of Groningen, Friesland, and Drenthe. Of the 135 schools, 122 decided to participate, and 3145 eligible adolescents at those schools, born between October 1, 1989, and September 30, 1991, were contacted for participation. After the recruitment process, 2229 adolescents (mean age ± SD = 11.1 ± 0.6 years) were included in the baseline survey (*T_1_*: March 2001-July 2002) and were followed up every 2 to 3 years until early adulthood. Cohort details can be found elsewhere.^28^ All respondents provided informed consent for study participation. Ethical approval was obtained from the Dutch Central Committee on Research Involving Human Subjects (approval no. NL38237.042.11).

**Figure 1:**
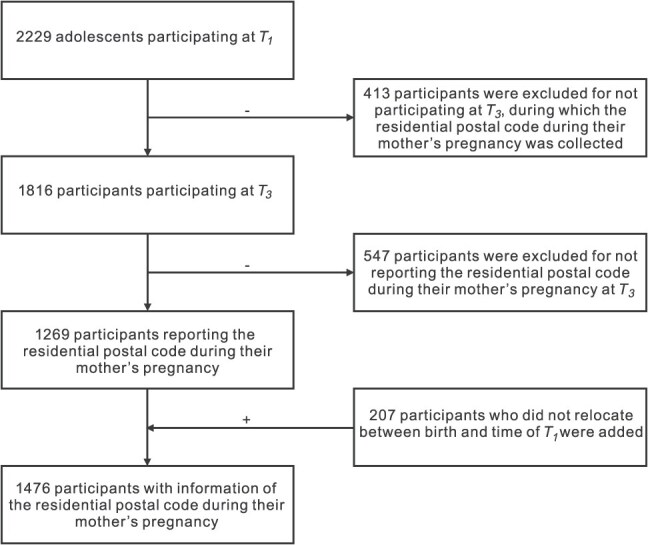
Selection of participants of the Tracking Adolescents’ Individual Lives Survey (TRAILS) cohort for a study of prenatal exposure to green space and mental health in early adolescence, 1989-2002.

In this study, we included 1476 participants; we excluded 753 participants without data on the residential postal code during their mothers' pregnancy, which was reported by the mother at the third survey wave (*T_3_*: September 2005-August 2007). As indicated in Figure 1, 413 individuals dropped out before *T_3_*; 547 individuals participated at *T_3_*, but their mothers did not report the residential postal code during pregnancy. We additionally included 207 individuals who did not relocate between birth and *T_1_*, combining the relocation frequency data assessed at *T_1_* and data on the postal code of residence at *T_1_*.

### Outcomes

Externalizing and internalizing problems are 2 broad-band, distinctive, and well-established dimensions of adolescent mental health problems.^29^ Externalizing problems are characterized as outer-directed issues, whereas internalizing problems refer to more inner-directed problems.^30^ These 2 indicators were measured at *T_1_* using the Youth Self Report.^31^ Externalizing problems (Cronbach’s α = .85) were measured through the mean score of the Aggressive and Delinquent Behaviors subscales comprising 32 items on a 3-point Likert scale (not true, somewhat or sometimes true, and very or often true). Internalizing problems (Cronbach’s α = .87) were assessed by averaging 31 items on the Withdrawn/Depressed*,* Anxious/Depressed*,* and Somatic Complaints subscales. Both scores range from 0 to 2. Higher scores indicate more externalizing or internalizing problems.

Substance use was operationalized through tobacco and alcohol use, assessed by 2 questions from the Antisocial Behavior Questionnaire at *T_1_*.^32^ Respondents were asked how often they had ever smoked cigarettes or drunk alcohol (never, once, 2-3 times, 4-6 times, and ≥7 times). Because responses of higher frequencies were rare (ie, 4.3% used tobacco and 14.6% used alcohol more than once), we dichotomized the variable into never and used.

### Mediators

Gestational age (weeks) and birth weight (pounds) were assessed by asking the mother, via the TRAILS Family History Interview at *T_1_*, how long the pregnancy lasted and how much the child weighed at birth.

### Green space exposure

Green space was measured using the satellite-based Normalized Difference Vegetation Index (NDVI) derived from Landsat5 and Landsat7 imagery obtained from the Google Earth Engine.^33^ The NDVI captures greenness based on top-of-atmosphere reflectance of visible red and near-infrared imagery bands with a 30 × 30 m resolution. To maximize exposure contrasts, we collected cloud-free NDVI images in the growing season (May to September), during which the NDVI quality was the highest, of the participants’ year of birth (ie, 1989-1991) and *T_1_* (ie, 2001-2002). Pixels with positive NDVI values were used, with higher values indicating more greenness (range, 0-1). Green space exposure during both prenatal (*T_0_*) and early adolescence (*T_1_*) periods were measured by averaging NDVI pixel values per 4-digit postal code area of residence at each time point.^34^^-^^36^

### Covariates

**Figure 2:**
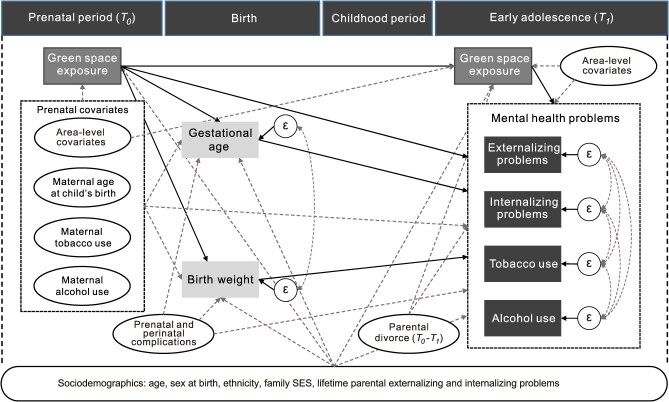
A path diagram depicting the hypothesized associations among exposures, mediators, outcomes, and covariates for a study of prenatal exposure to green space and mental health in early adolescence, 1989-2002. The path diagram resembles a causal directed acyclic graph (DAG) but differs in several important ways: it allows for two-headed arrows to represent unexplained covariance between variables, assumes a specific functional form (e.g., linear relationships) for the associations, and incorporates distributional assumptions for all endogenous variables in the structural equation model. A description of the path diagram—commonly used to construct structural equation modeling (SEM) frameworks, particularly in psychology—is included here, as this tool may be unfamiliar to epidemiologists. Clarifying its similarities and differences with causal directed acyclic graphs (DAGs) may aid readers in understanding its purpose and interpretation. Solid lines present the associations of interest; dash lines show the potential confounding paths we controlled for. ε denotes the residual variance of the corresponding variable. Green space exposure at *T_1_* was modeled to account for its shared variance with prenatal exposure to green space (*T_0_*). Likewise, both mediators (ie, gestational age and birth weight) were modeled simultaneously (with a correlational 2-headed arrow between their residuals) to control for their covariance. We did not specify any path between prenatal exposure to green space and prenatal and perinatal complications, due to limited evidence of their association (and the direction of the path), which was also supported by the model fit information indicating no important unspecified correlations. All covariates included were specified as exogenous variables with correlations among them implicitly accounted for. Area-level covariates include the area-level deprivation and social fragmentation indices. The interval of the “frame” for each period presented in the figure does not indicate the time interval of the corresponding period denoted in the frame. Abbreviation: SES, socioeconomic status.

We selected covariates based on their assumed associations with the exposures, mediators, and outcomes indicated in Figure 2. Appendix S1 provides in-depth covariate information. Briefly, we included sex at birth (female or male); age at *T_1_*; ethnicity (native or immigrant); family socioeconomic status (SES) at *T_1_*^37^; parental divorce between *T_0_* and *T_1_* (yes or no); lifetime parental externalizing and internalizing problems (measured at *T_1_*)^38^; maternal age at childbirth; maternal tobacco use during pregnancy (never use, < 1-10, or ≥ 11 cigarettes day^−1^); maternal alcohol use during pregnancy (never use, < 1-3, or ≥ 4 drinks week^−1^); prenatal and perinatal complications (no complication, 1-4, or > 4 complications); and neighborhood deprivation and social fragmentation (these 2 were measured at both *T_0_* and *T_1_*).^39^

### Statistical modeling

#### The main analysis

We fitted a structural equation model based on Figure 2 to assess the direct effects of prenatal exposure to green space (*T_0_*) on the 4 mental health outcomes (namely externalizing problems, internalizing problems, tobacco use, and alcohol use) and the indirect effects of prenatal exposure to green space (*T_0_*) on these outcomes via gestational age and birth weight. Structural equation models allowed us to (1) separate the estimation of the association between prenatal exposure to green space and mental health through different mediating paths while adjusting for covariance among multiple mediators^40^; (2) explicitly specify the assumed causal path from 1 factor to another (Figure 2); and (3) simultaneously estimate parameters for mediators and outcomes with different variable types (eg, continuous, binary), under several parametric assumptions (eg, linearity) and the assumption of no unmeasured exposure-mediator or outcome and mediator-outcome confounding.^41^^,^^42^ We assessed the proportion of the total effect mediated through each indirect path (ie, prenatal exposure to green space–gestational age–mental health; prenatal exposure to green space–birth weight–mental health) as the extent to which there is mediation. The path (green space (*T_0_*)–green space (*T_1_*)–mental health) was included only to control for the temporal intercorrelation between green space at both time points (to estimate the so-called controlled direct effects); “mediation” via this path was not of interest.^42^ We used the weighted least squares estimator to obtain probit regression coefficients for binary outcomes (ie, tobacco and alcohol use), and linear regression coefficients for continuous outcomes (ie, externalizing and internalizing problems) and mediators (ie, gestational age and birth weight). Given that the area-level (ie, 4-digit postal code area) variances for all outcomes were minor, we disregarded the area-level random intercepts for model parsimony.

We reported the standardized coefficient (β*_STDYX_*), interpreted as the SD-unit difference in the outcome or mediator per SD-unit difference in the mediator or exposure, and 95% CIs. For binary outcomes, the SD of the continuous latent response variable was used for standardization. We evaluated the model fit based on the root mean square error of approximation (<0.06), comparative fit index (> 0.95), Tucker-Lewis index (> 0.95), and standardized root mean residual (<0.08) following the recommended cutoff criteria.^43^ The analyses were conducted in Mplus 8.9.^44^

#### Missing data handling

A total of 94 respondents (6.4%) had missing data for 13 variables; the proportion of missing data for each variable ranged from 0.5% to 2.3%. Missing data were imputed using multiple imputations with Bayesian estimation by all variables in the model. Parameter estimates were computed and averaged over the 10 generated sets, using Rubin’s rules.^45^

#### The sensitivity analyses

To test the result robustness, first we assessed the potential for unmeasured confounding to the estimated associations using the E-value approach (Appendix S2).^46^ Second, we controlled for urbanicity (Appendix S1) to further assess the potential confounding by unmeasured urban-related factors.^47^ Third, we replaced urbanicity with fine particulate matter (PM_2.5_)^48^ and traffic noise^49^ (Appendix S1) in the model to test if confounding by urbanicity, if any, was driven by these 2 factors. Fourth, we used inverse probability weighting to account for the potential sample selection bias driven by the excluded sample (Appendix S3).^50^^-^^52^ Fifth, we refitted the model from the main analysis using prenatal exposure to green space assessed with seasonal differences in NDVI during pregnancy incorporated (Appendix S4). Finally, we conducted a complete case analysis (*n* = 1382) to assess if the data imputation affected our analytical results.

## Results

### Study sample

There were some differences in characteristics between the analytical (*n* = 1476) and excluded (*n* = 753) samples. Results indicated that the included participants were more likely to be of native origin, tended to have a longer length of gestation and higher birth weight and family SES, and were less likely to smoke cigarettes before age 11 years. These participants also were less likely to have divorced parents or parents with externalizing and internalizing problems, and their mothers were more likely to be older, drink more alcohol, or smoke fewer cigarettes during pregnancy (Table S1).

Of the 1476 participants in the analytical sample (Table 1), boys (51.4%) were slightly more often included than girls (48.6%); most adolescents were of native origin (93.7%), did not smoke (88.2%) or did not drink alcohol before *T_1_* (69.3%), and did not experience parental divorce (85.0%). More than half of mothers experienced prenatal or perinatal complications (59.0%; Appendix S1). Most mothers did not smoke (71.4%) or drink alcohol (79.2%) during pregnancy.

**Table 1:** Characteristics of the Tracking Adolescents’ Individual Lives Survey study participants and their parents during participants’ prenatal (1989-1991) and early adolescence (2001-2002) periods for a study of prenatal exposure to green space and mental health in early adolescence, the Netherlands.

**Variable**	**Data** ^a^
*Outcomes*	
Externalizing problems	0.27 ± 0.19
Missing data	24
Internalizing problems	0.36 ± 0.23
Missing data	34
Tobacco use	
Never	1288 (88.2)
At least once	173 (11.8)
Missing data	15
Alcohol use	
Never	1011 (69.3)
At least once	448 (30.7)
Missing data	17
*Mediators*	
Gestational age (weeks)	39.87 ± 1.85
Missing data	10
Birth weight (lb.)	6.84 ± 1.19
Missing data	24
*Demographics*	
Age at *T_1_* (years)	11.1 ± 0.5
Sex at birth	
Female	759 (51.4)
Male	717 (48.6)
Ethnicity	
Native	1383 (93.7)
Immigrant	93 (6.3)
Family socioeconomic status (*T_1_*)	0.05 ± 0.77
Missing data	8
Parental divorce between *T_0_* and *T_1_*	
Not divorced	1255 (85.0)
Divorced	221 (15.0)
Parental externalizing problems (*T_1_*)	0.11 ± 0.36
Missing data	13
Parental internalizing problems (*T_1_*)	0.51 ± 0.77
Missing data	16
No. of prenatal and perinatal complications	
None	601 (41.0)
1-4	647 (44.1)
>5	218 (14.9)
Missing data	10
Maternal age (years)	29.98 ± 4.31
Missing data	33
Maternal daily cigarette use	
Never	1048 (71.4)
1-10	335 (22.8)
≥11	84 (5.7)
Missing data	9
Maternal alcohol use, glasses week^−1^	
Never	1161 (79.2)
1-3	287 (19.6)
≥4	18 (1.2)
Missing data	10
Green space (*T_0_*)	0.42 ± 0.10
Deprivation (*T_0_*)	2.40 ± 2.39
Social fragmentation (*T_0_*)	2.16 ± 3.02
Green space (*T_1_*)	0.42 ± 0.09
Deprivation (*T_1_*)	2.04 ± 2.42
Social fragmentation (*T_1_*)	1.64 ± 2.79
Changes (absolute value) in green space (*T_0_-T_1_)*	0.05 ± 0.06

a Data are reported as no., no. (%), or mean ± SD.

### Results of the main analysis

**Figure 3:**
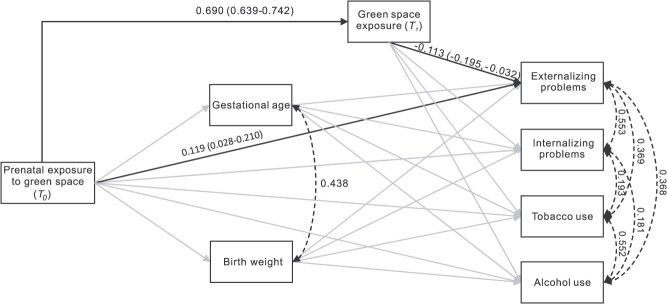
A path diagram presenting standardized associations (β*_STDYX_*) between prenatal exposure to green space (*T_0_*), green space exposure at *T_1_*, mediators (ie, gestational age, birth weight), and outcomes (externalizing problems, internalizing problems, tobacco use, and alcohol use) and 95% CIs, TRAILS cohort, 1989-2002. Black lines present associations with 95% CIs not including null; gray lines present associations with 95% CIs including null. The association β*_STDYX_* is interpreted as the SD unit difference in the outcome or mediator per SD unit difference in the mediator/exposure. For binary outcomes (tobacco and alcohol use), the SD of the continuous latent response variable was used for standardization. Gestational age was measured in weeks; birth weight was measured in pounds.

Figure 3 and Table S3 show the structural equation model results. The structural equation model had an excellent model fit (root mean square error of approximation = 0.013; comparative fit index = 0.998; Tucker-Lewis index = 0.982; standardized root mean residual = 0.012). The results showed that prenatal exposure to green space (*T_0_*) was not associated with either gestational age or birth weight, and these 2 variables were not associated with any mental health outcomes at *T_1_*. These results indicated no mediating roles of gestational age and birth weight on the associations between prenatal exposure to green space and mental health outcomes. We also found null direct associations between prenatal exposure to green space (*T_0_*) and mental health outcomes, except for externalizing problems, indicating that 1 SD more green space exposure prenatally was associated with a 0.119 SD (95% CI, 0.028-0.210) more externalizing problems at *T_1_*. In contrast, for green space exposure at *T_1_*, we found that adolescents with 1 SD more exposure at *T_1_* had a 0.113 SD (95% CI, −0.195 to −0.032) fewer externalizing problems at *T_1_*. Consistent with the minor area-level variance in the null model, both green space–externalizing problems associations were non-significant when modeling green space exposure at *T_0_* and *T_1_* separately.

### Results of the sensitivity analyses

First, the E-values for the point estimate and lower confidence bound of the observed association between prenatal exposure to green space (*T_0_*) and externalizing problems were 1.47 and 1.19, respectively, indicating a low risk of unmeasured confounding, which could fully explain this association (Appendix S2).^46^ Second, consistent with the E-values, adjusting for urbanicity only slightly attenuated the association between prenatal exposure to green space (*T_0_*) and externalizing problems, although such adjustment moved the CI to include null (β*_STDYX_* = .096; 95% CI, −0.003 to 0.195) (Table S4, Figure S1). In contrast, the association between exposure to green space (*T_1_*) and externalizing problems remained robust after adding urbanicity (β*_STDYX_* = −.116; 95% CI, −0.202 to −0.031) (Table S4, Figure S1). Third, controlling for PM_2.5_ may have induced multicollinearity issues in the associations between exposure to green space (both *T_0_* and *T_1_*) and externalizing problems, which showed similar point estimates but with CIs approximately 3 times wider than the counterparts in the main analysis (Table S4, Figure S1). This prevented us from assessing if PM_2.5_ could explain the observed associations between exposure to green space and externalizing problems. Controlling for noise did not change these associations markedly (Table S4, Figure S1). Fourth, adjusting for inverse probability of attrition weights^50^ only slightly changed the estimated association between prenatal exposure to green space and externalizing problems, indicating that sample selection bias to this estimate may be small (Appendix S3). Finally, the analysis using NDVI, accounting for seasons during pregnancy, and the complete case analysis, returned results similar to those of the main analysis (Tables S5 and  S6).

### Results of post hoc analyses

Because the model we fitted in the main analysis is statistically equivalent to the model including changes in green space exposure from *T_0_* to *T_1_* while controlling for green space exposure at *T_0_* or *T_1_* (Appendix S5), we speculated that both green space–externalizing problems associations observed in the main analysis are consequences of changes in exposure to green space between the 2 time points (~11 years apart). To test this notion, we fitted 2 models (model 2a and 2b; Appendix S5) that included the change score of exposure to green space from *T_0_* to *T_1_*. We controlled for prenatal exposure to green space at *T_0_* in model 2a, and exposure to green space at *T_1_* in model 2b. Externalizing problems was used as the outcome, and all covariates were controlled in both models (Appendix S5).

**Figure 4:**
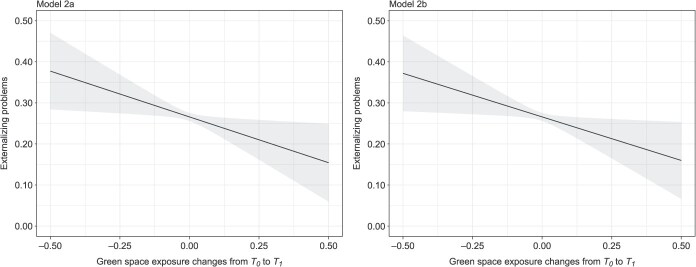
The unstandardized association between changes in green space exposure from *T_0_* to *T_1_* and externalizing problems at *T_1_*. Model 2a includes green space exposure at *T_0_* and changes in green space exposure from *T_0_* to *T_1_* (Appendix S5); model 2b includes green space exposure at *T_1_* and changes in green space exposure from *T_0_* to *T_1_* (Appendix S5). All covariates were controlled in both models as the main analysis. Positive values on the *x*-axis indicate increased green space exposure from *T_0_* to *T_1_*, and negative values indicate decreased exposure from *T_0_* to *T_1_*. The assumed mediators and other mental health outcomes that were not associated with green space exposure at *T_0_* and *T_1_* were removed, for model parsimony.

Model 2a and 2b had highly similar results on the estimated association between changes in green space exposure and externalizing problems, indicating that adolescents with increased green space exposure from *T_0_* to *T_1_* had fewer externalizing problems at *T_1_* than adolescents with decreased exposure, and stronger amounts of exposure increase (decrease) from *T_0_* to *T_1_* were associated with lower (higher) levels of externalizing problems at *T_1_* (Figure 4). Such associations were mainly driven by changes in green space exposure caused by residential relocation, because movers (*n* = 604; for exposure changes, mean ± SD = 0.090 ± 0.070) typically experienced more exposure changes than did nonmovers (*n* = 872; for exposure changes, mean ± SD = 0.025 ± 0.021). Furthermore, the associations for changes in green space exposure obtained from Model 2b and 2a were identical to the associations for green space exposure at *T_0_* (but in the opposite direction) and at *T_1_*, respectively, from the main analysis (Figure S2), which confirmed our speculation that both associations of exposure to green space with externalizing problems observed in the main analysis indicated the association between changes in green space exposure over childhood (from birth to early adolescence) and externalizing problems in early adolescence.

## Discussion

Our study assessed whether prenatal exposure to green space is associated with mental health in early adolescence. Contrary to our hypothesis, after controlling for green space exposure in early adolescence and multiple covariates, we found that more prenatal exposure to green space was associated with more externalizing problems in early adolescence. This association was not mediated by either gestational age or birth weight. In contrast, more green space exposure in early adolescence was associated with fewer externalizing problems in early adolescence.

Although contrary to our hypothesis, the observed positive association between prenatal exposure to green space and externalizing problems aligns with the findings of Maitre et al.,^17^ the only study, to our knowledge, that investigated this association. Those authors reported that children with more prenatal exposure to green space exhibited more externalizing problems at ages 6-11 years. Our sensitivity and post hoc analyses generated 2 possible explanations for the unexpected association we found. First, this association might suffer from residual confounding by unmeasured area-level confounders. We tested this possibility by additionally controlling for urbanicity, which attenuated the association between prenatal exposure to green space and externalizing problems and resulted in the association becoming statistically non-significant. This result attenuation could be attributed to the potential confounding by some urban-related factors that are negatively correlated with both green space exposure and externalizing problems. Such urban-related factors, for example, could be the health care services, which were less accessible in less urbanized areas in the Netherlands.^53^ Prior studies found that access to health care was essential to maternal and newborn health,^54^^-^^56^ and, in turn, could contribute to children’s later-life mental development.^27^ Thus, the observed positive association between prenatal exposure to green space and externalizing problems could be driven by the potentially higher risks of externalizing problems in adolescents whose mothers resided in areas with less access to health care services during pregnancy. Alternatively, some other urban-related factors, such as low walkability caused by poorer road connectivity and limited access to facilities,^57^^,^^58^ which may be typical in less urbanized areas, could be other candidate confounders. However, these speculations may require further testing, because urbanicity is only a crude proxy of multiple urban-related factors, and controlling for urbanicity only slightly attenuated the association between prenatal exposure to green space and externalizing problems (consistent with the E-value indicating a low risk of unmeasured confounding).

Second, the unexpected association between prenatal exposure to green space and externalizing problems in early adolescence could be the consequence of changes in green space exposure over childhood (from birth to early adolescence). Our post hoc analysis indicated that adolescents with increased green space exposure over childhood (mainly driven by residential relocation) had fewer externalizing problems in early adolescence. Childhood is a time when behaviors begin to form and are highly malleable.^59^ Some studies found that increased green space exposure, driven by residential relocation or neighborhood greenery interventions (eg, park renovations), could promote children’s physical activities,^60^^-^^63^ thereby protecting against externalizing problems.^64^ The pathway generated from these studies may explain our post hoc finding. Caution, however, is needed when interpreting this finding, because we assessed changes in green space exposure over childhood by only accounting for the exposure at the start (ie, prenatal) and end (ie, early adolescence) points of childhood. Such an assessment might be overly simplistic to capture the childhood exposure trajectory, especially for children moving multiple times, although this type of child is underrepresented (16.6%) in our sample. Moreover, such moves could also be self-selected because parents often move to a place suitable for raising children (eg, more greenery) after childbirth.^65^ This selective move, for example, may occur more often with the first child than the second or third child and more often among higher-income families who can afford such movement.^65^^-^^67^ However, our observed E-value of 1.47 indicated that the risk of severe residual confounding (eg, by self-selection) that could fully explain our estimated associations away is low,^43^ and we controlled for multiple covariates identified as important confounders in previous literature.^16^^-^^18^

We observed a disparity in the estimated association between prenatal exposure to green space and externalizing problems before and after adjusting for green space exposure in early adolescence, although, somewhat surprisingly, this association was null before such adjustment and became significantly positive after adjustment. Even so, it might still be necessary to isolate prenatal exposure from its correlation with the exposure after birth in the exploration of sensitive exposure periods. Such necessity, however, has rarely been considered by previous relevant studies. Pagalan et al.^16^ only incorporated prenatal exposure, whereas Maitre et al.^17^ modeled prenatal and childhood exposure separately without controlling for their covariance. A study in Shanghai incorporated both prenatal and postnatal exposures, but the strong exposure correlation, possibly due to residential immobility over time, led to a multicollinearity issue that caused unstable estimates.^18^ In these studies, the mental health effects of exposures after birth might add up to the estimated effect for prenatal exposure due to the omission of temporal intercorrelations among the exposures. In our study, we may have obtained an association for changes in green space exposure from birth to early adolescence after jointly modeling green space exposure at these 2 time points (as indicated in our post hoc analysis). Thus, differences in exposure periods incorporated should be considered when comparing previous findings with ours.

We found that gestational age and birth weight did not mediate the prenatal green space-mental health associations. Specifically, these 2 birth outcomes were not associated with either prenatal exposure to green space or mental health in early adolescence. One explanation is that our sample only included few cases of adolescents with preterm birth (gestational age < 37 weeks) and low birth weight (<2500 g), and such low variability of these variables might prevent us from obtaining robust associations between them and both the exposure and outcomes. The mechanism of the association between prenatal exposure to green space and later-life mental health has also been assessed by other studies that focused exclusively on mediation by air pollution, although no clear evidence of such mediation was observed.^16^^,^^18^ However, we did not assess this, because of the high correlation between green space and air pollution we observed. This high correlation, not observed by Pagalan et al.^16^ and Chen et al.,^18^ could be attributed to the low resolution (1 × 1 km) of the air pollution data we used, although this data was frequently used previously.^68^^,^^69^ Data with higher resolution were unavailable over the prenatal period used in our study.

The present study extends existing research in 3 ways. First, we incorporated green space exposures before and after birth, allowing us to assess whether each time-specific exposure acts independently on the outcome. Second, we included multiple mental health outcomes that showed the green space–mental health association might differ across outcomes. Third, we incorporated multiple (potential) confounders over the early life course to facilitate the robustness of the analytical results.

We also acknowledge several limitations. First, we only controlled for green space exposure in early adolescence, which may limit us with regard to distinguishing prenatal exposure to green space from its intercorrelations with the exposures at multiple time points after birth. Second, as in prior studies,^16^^-^^18^ we did not account for residential mobility during the mother's pregnancy when assessing prenatal green space exposure, which might have affected our effect estimates, although some evidence indicated that such bias might be minor.^70^^-^^72^ Third, we used the NDVI measure to assess the overall greenness exposure, which may not capture the green space–mental health association potentially driven by certain features of greenness (eg, aesthetics, accessibility).

In conclusion, this study investigated the associations between prenatal exposure to green space and mental health problems in early adolescence. We observed a positive association between prenatal green space exposure and externalizing problems in early adolescence. There are 2 possible explanations for this result. First, controlling for urbanicity attenuated this association to become non-significant, but the extent of its attenuation was minor. Second, this association might be a consequence of changes in green space exposure over childhood (from birth to early adolescence), indicating that individuals with increased green space exposure over childhood had fewer externalizing problems in early adolescence. We found null associations between prenatal exposure to green space and other outcomes, and no evidence of mediation by gestation age and birth weight. Future studies could build on our findings by adjusting for more-specific urban factors and incorporating green space exposure at multiple time points across the entire early life course.

## Acknowledgments

The authors thank Dr. Ellen Hamaker, Dr. Beth Grandfield, and Dr. Kyle Lang for their help with the statistical analysis. This research is part of the Tracking Adolescents’ Individual Lives Survey (TRAILS). Participating centers in TRAILS include various departments of the University Medical Centre and University of Groningen, Utrecht University, the Radboud University Medical Centre, and the Parnassia Psychiatric Institute, all in the Netherlands. The authors thank all TRAILS members who participated in this research or worked on this project to make it possible. This study was presented at the International Medical Geography Symposium, Atlanta, GA, July 14-19, 2024.

## Supplementary Material

Web_Material_kwae373

## Data Availability

The TRAILS survey data are available at https://www.trails.nl/en/hoofdmenu/data/data-use, subject to the approval of the TRAILS committee and data manager. The authors have no right to share the TRAILS data.
