# Supplementary material for: Prenatal exposure to green space and mental health in early adolescence: findings from the TRAILS study
Source: Am J Epidemiol. 2024 Sep 24;194(7):1949–58. doi: 10.1093/aje/kwae373 (PMC12234223; doi:10.1093/aje/kwae373)
Supplement: Web_Material_kwae373 [file web_material_kwae373.zip › supplementary_materials_FINAL_with title page.docx]

**Supplemental Material**

**Prenatal exposure to green space and mental health in early adolescence: Findings from the TRAILS study**

Yi Zeng, Gonneke W. J. M. Stevens, Tomáš Paus, & Marco Helbich

**Table of contents**

**Appendix S1.** In-depth information on some covariates.

**Appendix S2.** The sensitivity analysis for evaluating the potential for unmeasured confounding.

**Appendix S3.** The sensitivity analysis for evaluating and correcting sample selection bias.

**Appendix S4.** The assessment of prenatal green space exposure considering seasonal differences in NDVI.

**Appendix S5.** The model conversion.

**Table S1.** Results of the attrition analysis.

**Table S2.** Bivariate correlations for the study variables.

**Table S3.** Results of the main analysis.

**Table S4.** Results of the sensitivity analyses to adjusting for urbanicity, PM_2.5_, or traffic noise.

**Table S5.** Results of the model using prenatal green space exposure assessed with seasonal differences in NDVI during pregnancy incorporated.

**Table S6.** Results of the complete case analysis.

**Figure S1.** Associations of green space at *T_0_* and *T_1_* with externalizing problems, internalizing problems, tobacco use, and alcohol use from models with different area-level factors included.

**Figure S2.** Unstandardized results of the post-hoc analysis.

# **Appendix S1.** In-depth information on some covariates.

*Ethnicity* of adolescent participants was identified based on their immigrant family history recorded at *T_1_*. Specifically, adolescents were identified as immigrants if at least one of their parents was born in a non-Western country including Turkey, Morocco, Surinam, Dutch Antilles, Indonesia, Islamic countries (e.g., Iraq, Iran, Somalia), or others; the rest of adolescents were identified as native. ^1^

*Family socioeconomic status* (SES) was assessed at *T_1_* using five indicators, including both parents’ education levels, occupation levels, and household income based on the International Standard Classification of Occupations. ^2^ All these indicators were averaged after standardization. We controlled this variable as a potential confounder for the association between prenatal green space and mental health outcomes at *T_1_* under the assumption that family SES is time-invariant from the prenatal period to early adolescence. We believe this is a reasonable assumption given the strong correlation (0.85) between family SES at *T_1_* (around age 11 years) and *T_4_* (around age 19 years).

*Lifetime parental externalizing and internalizing problems before T_1_* were measured based on several questions assessing professional treatment, medication use, and lifetime occurrence of five dimensions of psychopathology, including depression, anxiety, substance abuse, antisocial behaviors, and psychoses, by means of the TRAILS Family History Interview at *T_1._* ^3^ The domain of lifetime externalizing problems was constructed based on scores of substance abuse and antisocial behaviors. The index of lifetime internalizing problems was measured by combining depression and anxiety. We used these two indices to indicate the familial vulnerability to these two problems, respectively.

*Maternal tobacco use during pregnancy* was measured at *T_1_* by asking the mother if and how much they smoked cigarettes during pregnancy (‘never’, ‘<1 cigarette/day’, ‘1-10 cigarettes/day’, ’11-20 cigarettes/day’, ‘1-2 packs/day’, and ‘>2 packs/day’). Due to the rare responses of higher frequencies, we grouped the variable into (no tobacco use, <1-10 cigarettes/day, or ≥11 cigarettes/day) by combining the high-frequency answers following Buschgens, et al. ^4^ and Marceau, et al. ^5^

*Maternal alcohol use during pregnancy* was measured at *T_1_* by asking the mother if and how much they drank alcohol during pregnancy (‘never’, ‘<1 glass/week’, ‘1-3 glasses/week’, ’4-10 glasses/week’, ‘10-20 glasses/week’, and ‘>20 glasses/week’). Due to the rare responses of higher frequencies, we grouped the variable into (no alcohol use, <1-3 drinks/week, or ≥4 drinks/week) by combining the high-frequency answers following Marceau, et al. ^5^

*The score of prenatal and perinatal complications* was composed by adding the scores for the degree of difficulties during pregnancy and delivery (each rated on a four-point scale: 0 = not at all, 1 = somewhat, 2 = fairly, 3 = very) and scores counting the presence of pregnancy problems (i.e., physical, social, or psychological problems), delivery problems (i.e., took a long time, complications, cesarean section, tongs or suction cup, and others), birth complications (i.e., lack of oxygen, staying in incubator, jaundice, blood transfusion, seizures or convulsions, congenital abnormalities, and others), the mother’s conditions after delivery (physical problems related to pregnancy, physical problems not related to pregnancy, maternity bed psychosis, and others) assessed at *T_1_*. We grouped this variable into three categories (no complication, 1-4 complications, or >4 complications), following Buschgens, et al. ^4^ Detailed information on each complication can be found in the table below. Information from this table shows that the composite score of prenatal and perinatal complications was mainly driven by some complications including the overall degree of difficulties during pregnancy, delivery, or birth, while the proportions of others, including mothers or newborns having other complications, such as seizures and congenital abnormalities, were minor.

Scale and distribution of items of prenatal and perinatal complications.

| **Items of prenatal and perinatal complications** | **Scale** | **Frequency** |
| --- | --- | --- |
| The degree of difficulties during pregnancy | Not at all = 0 | 1099 (74.9%) |
|  | Somewhat = 1 | 367 (25.0%) |
|  | Fairly = 2 | 1 (0.0%) |
|  | Very = 3 | Null |
| The degree of difficulties during delivery | Not at all = 0 | 891 (60.7%) |
|  | Somewhat = 1 | 575 (39.2%) |
|  | Fairly = 2 | 1 (0.0%) |
|  | Very = 3 | Null |
| *The presence of pregnancy problems:* |  |  |
| Physical problems related to pregnancy or birth | Yes = 1; No = 0 | 296 (20.2%); 1172 |
| Physical problems not related to pregnancy or birth | Yes = 1; No = 0 | 34 (2.3%); 1434 |
| Social problems | Yes = 1; No = 0 | 38 (2.6%); 1430 |
| Psychological problems | Yes = 1; No = 0 | 33 (2.2%); 1435 |
| *The presence of delivery problems:* |  |  |
| Took a long time | Yes = 1; No = 0 | 213 (14.5%); 1255 |
| Complications (e.g., breech presentation, umbilical cord around the neck, stool in amniotic fluid) | Yes = 1; No = 0 | 104 (7.1%); 1364 |
| Cesarean section | Yes = 1; No = 0 | 110 (7.5%); 1358 |
| Tongs or suction cup | Yes = 1; No = 0 | 146 (9.9%); 1322 |
| Others | Yes = 1; No = 0 | 229 (15.6%); 1239 |
| *Birth complications* |  |  |
| Lack of oxygen | Yes = 1; No = 0 | 36 (2.4%); 1432 |
| Staying in incubator | Yes = 1; No = 0 | 93 (6.3%); 1375 |
| Jaundice | Yes = 1; No = 0 | 81 (5.5%); 1387 |
| Blood transfusion | Yes = 1; No = 0 | 8 (0.5%); 1460 |
| Seizures or convulsions | Yes = 1; No = 0 | 3 (0.2%); 1465 |
| Congenital abnormalities | Yes = 1; No = 0 | 2 (0.1%); 1466 |
| Others | Yes = 1; No = 0 | 227 (15.5%); 1241 |
| *The mother’s conditions after delivery:* |  |  |
| Physical problems related to pregnancy or birth | Yes = 1; No = 0 | 204 (13.9%); 1264 |
| Physical problems not related to pregnancy or birth | Yes = 1; No = 0 | 19 (1.3%); 1449 |
| Maternity bed psychosis | Yes = 1; No = 0 | Null |
| Others | Yes = 1; No = 0 | 93 (6.3%); 1375 |

*Neighborhood deprivation* was compiled based on administrative register data of all residents over the Netherlands residing in each PC4 area at *T_0_* and *T_1_*. ^6^ Deprivation was measured by the reverse-coded standardized median household income, the share of households below the poverty line, and the unemployment rate. Input variables were z-scored and summed for each composite measure separately using data from 2005 since when the input data were available. Higher scores indicate more deprivation.

*Social fragmentation* was compiled based on administrative register data of all residents residing in each PC4 area at *T_0_* and *T_1_*. ^6^ Social fragmentation was assessed by the percentage of single-person households, unmarried adults, and newly relocated residents last year. Input variables were z-scored and summed for each composite measure separately using data from 2005 since when the input data were available. Higher scores indicate more social fragmentation.

*Urbanicity* was assessed using the address density per PC4 per year across the study period. Due to strong green space-urbanicity correlations at *T_0_* (*r*=-0.84) and *T_1_* (*r*=-0.80), we categorized the degree of urbanicity into five strata: not urbanized (<500 addresses/km^2^), hardly urbanized (500-1,000 addresses/km^2^), moderately urbanized (1,000-1,500 addresses/km^2^), strongly urbanized (1,500-2,000 addresses/km^2^), and extremely urbanized (≥2,000 addresses/km^2^) to reduce its correlation with green space at *T_0_* and *T_1_*.

*PM_2.5_* (fine particulate matter) was measured using ground-level estimates (1 × 1 km resolution) derived from the European Regional Estimates (V4.EU.03) in 2000. This estimate was based on chemical transport modeling on satellite data and calibrated using ground-level observations from monitor instruments in the European areas. Modeling details can be found elsewhere. ^7^ We categorized PM_2.5_ into quartiles also to reduce its strong correlation with green space at *T_0_* (*r*=-0.81) and *T_1_* (*r*=-0.88), although the correlations remained strong after the categorization (*r*=-0.76 and -0.83).

The *noise* layer (25 × 25 m resolution; dB(A)) in 2000, estimated by the Netherlands Environmental Assessment Agency, ^8^ reflects emissions of noise pollution from roads, railroads, and air traffic. The model used for estimation accounted for factors such as road traffic intensity, pavement type, noise barriers, train equipment, timetable for railways, types of rails, the number of flights with aircraft per job, size, type, and age of the aircraft, times of take-off and landing and acquisition, and flight paths (both horizontal and vertical). The daily average noise level was measured based on the day (7-19h), evening (19-23h; 5 dB(A) added for penalty), and night (23-7h; 10 dB(A) added for penalty).

# **Appendix S2.** The sensitivity analysis for evaluating the potential for unmeasured confounding.

We used the E-value approach to evaluate the robustness of the association between prenatal green space exposure and externalizing problems in early adolescence, obtained from the main analysis, to the potential unmeasured confounding bias. This approach assessed the minimum strength of the association that would be required for an unmeasured confounder with both the exposure (prenatal green space) and outcome (externalizing problems), conditional on the observed covariates, to explain away the estimated exposure-outcome association. ^9^ Given that the calculation of E-value is derived from the association on the risk ratio (*RR*) scale, we first converted our estimated association (*d*), which indicated the mean difference (standard deviation unit difference) in a continuous outcome between individuals with a standard deviation unit difference in the exposure, to *RR* using *Equation 1*, with approximate 95% confidence interval (based on the standard error: *S_d_*) using *Equation 2* ^9^. Afterwards, we applied the E-value formulas (*Equation 3a* and *3b*) to the converted *RR* and the limit of confidence interval closest to the null ^9^.

*Equation 1*: $RR\approx exp(0.91\times d)$

*Equation 2*: $95\%CI\approx(\exp\left\{ 0.91 \times d-1.78\times s_{d} \right\},exp\left\{ 0.91 \times d+1.78\times s_{d} \right\})$

*Equation 3a*: $E\mathrm{value}=RR+sqr\left\{ RR\times\left( RR-1 \right) \right\};if RR>1$

*Equation 3b*: $E\mathrm{value}=1/RR+sqr\left\{ 1/RR\times\left( 1/RR-1 \right) \right\};if RR<1$

In the main analysis, the observed association (*d*) between prenatal green space exposure and externalizing problems was 0.119 with an *S_d_* of 0.046. We converted these values to *RR* as 1.11 with the lower confidence interval of 1.03. Using the above-mentioned equations (*3a and 3b*), we obtained an E-value of 1.47 for the point estimate and an E-value of 1.19 for the lower limit of the confidence interval. These two E-values could also be presented as 0.42 and 0.19 by converting *RR* back to *d* using *Equations 1 and 2*.

The E-value for the point estimate indicated that the observed association of 0.119 (*RR*: 1.47) could be fully explained away by an unmeasured confounder having an association at least as large as 0.42 (*RR*: 1.47) with both the exposure and the outcome, conditional on the observed covariates, but weaker confounding could not do so. Given that this association (E-value) is much larger than the association for any other covariates with externalizing problems (see the table below and other studies, such as Maitre, et al. ^10^), it seems implausible that we omitted a confounder having an association (with both prenatal green space exposure and externalizing problems) with an effect size larger than this E-value (0.119, *RR*: 1.47). Moreover, the E-value (0.19, *RR*: 1.19) for the lower confidence interval limit was the minimum strength of the association that the potential unmeasured confounder should have with both prenatal green space and externalizing problems, conditional on the observed covariates, to move the confidence interval to include the null. This E-value may still be a large threshold for the potential unmeasured confounder when comparing this value with the association between other covariates and externalizing problems (see the table below). Thus, we concluded that the risk of unmeasured confounders that could fully explain our estimated prenatal green space-externalizing problems association away might be low.

Estimated associations between covariates and externalizing problems

|  | Point estimate | SE | *P-value* |
| --- | --- | --- | --- |
| Green space (*T_0_*) | 0.119 | 0.046 | 0.011 |
| Green space (*T_1_*) | -0.113 | 0.041 | 0.006 |
| Gestational age | -0.015 | 0.030 | 0.622 |
| Birthweight | 0.053 | 0.030 | 0.075 |
| Family SES | 0.028 | 0.030 | 0.347 |
| Parental externalizing problems | 0.006 | 0.028 | 0.830 |
| Parental internalizing problems | 0.025 | 0.027 | 0.363 |
| Maternal age at childbirth | -0.039 | 0.028 | 0.160 |
| Maternal tobacco use during pregnancy | 0.071 | 0.026 | 0.006 |
| Maternal alcohol use during pregnancy | 0.004 | 0.029 | 0.898 |
| Prenatal and perinatal complications | -0.015 | 0.027 | 0.589 |
| Age at *T_1_* | 0.067 | 0.028 | 0.015 |
| Sex (Ref = female) | 0.197 | 0.026 | 0.000 |
| Ethnicity (Ref = native) | 0.001 | 0.027 | 0.958 |
| Parental divorce (Ref = not divorced) | 0.027 | 0.028 | 0.321 |
| Deprivation (*T_0_*) | -0.033 | 0.041 | 0.416 |
| Social fragmentation (*T_0_*) | 0.080 | 0.058 | 0.166 |
| Deprivation (*T_1_*) | 0.094 | 0.045 | 0.035 |
| Social fragmentation (*T_1_*) | -0.131 | 0.054 | 0.015 |

Note: *T_0_*: prenatal period; *T_1_*: early adolescence

# **Appendix S3.** The sensitivity analysis for evaluating and correcting the sample selection bias

Given that the retained and excluded sample differed in some characteristics (Table S1), selective attrition bias that the retained sample may no longer be a faithful presentation of the original sample may be induced, thereby possibly affecting the validity and generalizability of the exposure-response estimation. In light of this, we used inverse probability weighting (IPW) to evaluate and correct (if any) for such selective attrition bias in the estimated association between prenatal green space and externalizing problems. Two separate approaches were developed for this.

1. We used inverse-probability-of-attrition weighting (IPAW) to adjust for differences in the average value of certain covariates between the retained and excluded samples. ^11-14^ Specifically, we fitted a logistic regression to model and estimate the probability of being selected into the analytical sample, conditional on the observed covariates which showed significant differences in Table S1. Further, we simplified the model by only including those (i.e., ethnicity, family socioeconomic status (SES), parental divorce, maternal age at childbirth, adolescents’ gestational age, and adolescent tobacco use at age 11 years) showing significant associations (*p-value<*0.05) with the sample attrition in the fitted logistic regression model. ^15^ An IPA-weight for the observation for each participant was calculated by the inverse of the probability obtained from the aforementioned logistic regression with an additional term to stabilize the weight. Assigning the IPA-weight to the analytical sample created a pseudo-sample that could be viewed as a randomly selected sample from the original sample. The stabilized IPA-weight was defined as:

*Equation a*: ${SW}_{i}^{S}=\frac{P[S_{i}=1]}{P[S_{i}=1|z_{i}^{S}]}$

Where ${SW}_{i}^{S}$ is the IPA-weight for the participant $i$; $P[]$ is the marginal/conditional probability function; $S_{i}=1$ if the participants $i$ was selected into the study; $z_{i}^{S}$ is a vector containing the selected covariates (as aforementioned) for the participant $i$. The denominator of *equation a* represents the probability of being selected into the analytical sample conditional on the values of covariates $z_{i}^{S}$. The numerator of *equation a*, a marginal probability of being selected into the analytical sample, was used to stabilize the weight.

Afterwards, we fitted an IPA-weighted linear regression to assess the association between prenatal green space exposure and externalizing problems in early adolescence. All covariates included in the main analysis were also included in this regression model since incorporating the IPA-weight did not adjust for the potential confounding of these covariates on the exposure-outcome association in the regression model.

As the table shown below, we observed a similar positive association of prenatal green space exposure with externalizing problems in early adolescence between both the unweighted and IPA-weighted regression model, with the point estimate slightly stronger in the estimate from the IPA-weighted regression [the unstandardized estimate: 0.232, (95% CI: 0.028, 0.435)] than the estimate from the unweighted regression model [0.208, (95% CI: 0.023, 0.393)].

Associations between covariates and sample attrition and the weighted association between prenatal green space exposure and externalizing problems

| Variables | Point estimate of the association | 95% confidence interval |
| --- | --- | --- |
| *Outcome: sample selection* |  |  |
| Ethnicity (ref: native) | -1.076 | -1.384, -0.772 |
| Family SES | 0.200 | 0.066, 0.334 |
| Parental divorce (ref: not divorced) | -0.790 | -1.024, -0.555 |
| Maternal age at childbirth | 0.072 | 0.049, 0.095 |
| Adolescent tobacco use at *T_1_* | -0.298 | -0.570, -0.023 |
| Adolescents’ gestational age | 0.065 | 0.018, 0.112 |
| *Outcome: externalizing problems* |  |  |
| Prenatal green space (unweighted) | 0.208 | 0.023, 0.393 |
| Prenatal green space (IPA-weighted: mean (SD): 1.0 (0.32)) | 0.232 | 0.028, 0.435 |

Note: *T_1_*: early adolescence; the point estimate of the association and 95% confidence interval were unstandardized; for the model regressing externalizing problems on prenatal green space exposure, we used the complete-case sample (*n*=1,382) and excluded gestational age and birthweight (two assumed mediators) with the adjustment of all covariates.

1. Instead of fitting an IPA-weight regression with covariates included as *approach 1*, we created an IP-weight to simultaneously correct for confounding of the observed covariates and selective sample attrition bias ^13,16^. Specifically, the IP-weight ${SW}_{i}$ for the participant $i$ is simply the product of IPA-weight ${SW}_{i}^{S}$, obtained from *equation a*, and the inverse-probability-of-exposure (IPE) weight ${SW}_{i}^{X}$, obtained from *equation b* (below); that is, ${SW}_{i}={SW}_{i}^{S}\times{SW}_{i}^{X}$. The IPE-weight adjusted for potential confounding of the observed covariates on the exposure-outcome association. Formally, the IPE-weight was defined as

*Equation b*: ${SW}_{i}^{X}=\frac{f[X_{i}|S_{i}=1]}{f[X_{i}|z_{i}^{X}, S_{i}=1]}$

Where the denominator is the conditional density function of exposure $X$ (prenatal green space) evaluated at the observed value of exposure $X_{i}$ for the participant $i$ in the retained sample $S_{i}=1$, given the values of a set of covariates $z_{i}^{X}$(all covariates included in the regression in the *approach 1* and the main analysis); the numerator is the marginal density function of $X$ evaluated at the observed value of exposure $X_{i}$ for the participant $i$ in the retained sample $S_{i}=1$, which was used for weight stabilization. Assigning the IPE-weight (${SW}_{i}^{X}$) to the analytical sample created a pseudo-sample in which the covariates incorporated in the weighting process were no longer correlated with the exposure of interest, which thereby adjusted for the confounding of these covariates on the estimation of the exposure-outcome association in the following analysis.

Afterwards, we fitted an IP-weighted linear regression to assess the association between prenatal green space exposure and externalizing problems with only the exposure and the outcome included. We observed a slightly stronger positive association between prenatal green space exposure and externalizing problems in early adolescence in the IP-weighted regression [the unstandardized estimate: 0.287, (95% CI: 0.028, 0.054)] than the association observed in the unweighted [0.208, (95% CI: 0.023, 0.393)] and IPA-weighted model [0.232, (95% CI: 0.028, 0.435)].

Associations between covariates and sample attrition and the weighted association between prenatal green space exposure and externalizing problems

| Variables | Point estimate of the association | 95% confidence interval |
| --- | --- | --- |
| *Outcome: externalizing problems* |  |  |
| Prenatal green space (unweighted) | 0.208 | 0.023, 0.393 |
| Prenatal green space (IPA-weighted: mean (SD): 1.0 (0.3)) | 0.232 | 0.028, 0.435 |
| Prenatal green space (IP-weighted: mean (SD): 1.1 (3.1)) | 0.287 | 0.028, 0.054 |

Note: The point estimate of the association and 95% confidence interval were unstandardized; for all three models regressing externalizing problems on prenatal green space exposure, we used the complete-case sample (*n*=1,382) and excluded gestational age and birthweight (two assumed mediators); we adjusted for all covariates in the unweighted and IPA-weighted models, while these covariates were incorporated into the IPE-weighting process in the IP-weighted model.

Overall, the similar estimate of the association for prenatal green space with externalizing problems between the unweighted and both weighted models may indicate a low risk of selective attrition bias in the estimated association obtained from the main analysis.

# **Appendix S4.** The assessment of prenatal green space exposure considering seasonal differences in NDVI.

We calculated the NDVI value for each season within each neighborhood and assigned the season-specific values to the participants based on the season of birth (e.g., individuals born in spring were assigned the average NDVI value in spring, winter, and autumn). The season was defined based on the astronomical definition: Spring (21/03 - 20/06), Summer (21/06 - 20/09), Autumn (21/09 - 20/12), Winter (21/12 - 20/03). We could not, however, assess the season-specific NDVI value for the participants based on the specific year of birth (1989, 1990, or 1991) since a large number of pixels have missing data, possibly due to extensive cloud cover and fewer images available in the early years, were found in the NDVI images in some seasons (especially in autumn and winter) in some years (details can be found in the table below).

|  | Neighborhoods with NA pixels > 30% | Neighborhoods with NA pixels > 50% | Neighborhoods with NA pixels > 70% | Neighborhoods with NA pixels > 90% |
| --- | --- | --- | --- | --- |
| Spring |  |  |  |  |
| 1989 | - | - | - | - |
| 1990 | - | - | - | - |
| 1991 | 30.8% | 28.4% | 24.0% | 18.4% |
| Summer |  |  |  |  |
| 1989 | 6.4% | 4.8% | 4.0% | 3.6% |
| 1990 | 11.6% | 3.6% | 0.8% | 0.4% |
| 1991 | 2.0% | 0.4% | - |  |
| Autumn |  |  |  |  |
| 1989 | 86.0% | 85.2% | 84.0% | 82.8% |
| 1990 | 3.2% | 0.8% | 0.8% | 0.8% |
| 1991 | 24.0% | 20.4% | 16.4% | 14.8% |
| Winter |  |  |  |  |
| 1989 | 9.6% | 7.6% | 6.0% | 5.2% |
| 1990 | 5.2% | 1.2% | - |  |
| 1991 | 40.0% | 26.4% | 18.8% | 14.8% |

To ensure a sufficient quality of NDVI in each season, we calculated the season-specific NDVI value by taking the mean value of NDVI for each pixel (30×30m) of the satellite images across 1989, 1990, and 1991 in the corresponding season and, as such ignored temporal changes in NDVI across these three years. We believe this is a reasonable assumption due to the high agreement of NDVI in a season across years except for the NDVI in the seasons with large missing values (details can be found in tables below).

Pairwise correlation of NDVI in a season across years.

*Spring*

|  | Spring_mean* | Spring_1989 | Spring_1990 | Spring_1991 |
| --- | --- | --- | --- | --- |
| Spring_mean* | 1 | 0.97 | 0.98 | 0.92 |
| Spring_1989 |  | 1 | 0.95 | 0.90 |
| Spring_1990 |  |  | 1 | 0.93 |
| Spring_1991 |  |  |  | 1 |

Note: Spring_mean* was calculated by taking the mean value of each NDVI pixel of the satellite images across 1989, 1990, and 1991 in spring.

*Summer*

|  | Summer_mean* | Summer_1989 | Summer_1990 | Summer_1991 |
| --- | --- | --- | --- | --- |
| Summer_mean* | 1 | 0.93 | 0.92 | 0.96 |
| Summer_1989 |  | 1 | 0.83 | 0.90 |
| Summer_1990 |  |  | 1 | 0.84 |
| Summer_1991 |  |  |  | 1 |

Note: Summer_mean* was calculated by taking the mean value of each NDVI pixel of the satellite images across 1989, 1990, and 1991 in summer.

*Autumn*

|  | Autumn_mean* | Autumn_1989 | Autumn_1990 | Autumn_1991 |
| --- | --- | --- | --- | --- |
| Autumn_mean* | 1 | 0.64 | 0.94 | 0.94 |
| Autumn_1989 |  | 1 | 0.50 | 0.57 |
| Autumn_1990 |  |  | 1 | 0.80 |
| Autumn_1991 |  |  |  | 1 |

Note: Autumn_mean* was calculated by taking the mean value of each NDVI pixel of the satellite images across 1989, 1990, and 1991 in autumn.

*Winter*

|  | Winter_mean* | Winter_1989 | Winter_1990 | Winter_1991 |
| --- | --- | --- | --- | --- |
| Winter_mean* | 1 | 0.95 | 0.91 | 0.82 |
| Winter_1989 |  | 1 | 0.82 | 0.82 |
| Winter_1990 |  |  | 1 | 0.68 |
| Winter_1991 |  |  |  | 1 |

Note: Winter_mean* was calculated by taking the mean value of each NDVI pixel of the satellite images across 1989, 1990, and 1991 in Winter.

Strong correlations of NDVI across seasons in our study.

|  | Spring_mean | Summer_mean | Autumn_mean | Winter_mean |
| --- | --- | --- | --- | --- |
| Spring_mean | 1 | 0.83 | 0.76 | 0.92 |
| Summer_mean |  | 1 | 0.80 | 0.84 |
| Autumn_mean |  |  | 1 | 0.81 |
| Winter_mean |  |  |  | 1 |

In the fourth sensitivity analysis, we replaced the original variable of prenatal green space exposure (in the main analysis) with the exposure accounting for the season-specific NDVI value during pregnancy in the regression model. In addition, we controlled for dummies of the season at birth to mitigate the confounding bias caused by the season-related confounders.

# **Appendix S5.** The model conversion.

As the figure below, Model 1 is the outcome model (i.e., the model including externalizing problems as the outcome) that we fitted in the main analysis. The process indicated below showed that Model 1 is statistically equivalent to Model 2a, which includes green space exposure changes from *T_0_* to *T_1_* and green space exposure at *T_0_*, and Model 2b, which includes green space exposure changes from *T_0_* to *T_1_* and green space exposure at *T_1_*. By comparing the coefficients of Model 1 with Model 2a and 2b, we observed that the coefficients for green space at *T_0_* (i.e., $\beta_{0}$) and *T_1_* (i.e., $\beta_{1}$) were, respectively, identical to the coefficients for green space exposure changes from *T_0_* to *T_1_* in Model 2b (with a different direction; -$\beta_{0}$) and Model 2a ($\beta_{1}$). This implies that both coefficients for green space exposure that we observed in the main analysis could be interpreted as the association between green space exposure changes from *T_0_* to *T_1_* and externalizing problems at *T_1_*. This might be further supported by the similar effect sizes (but with a different direction) of both coefficients (for green space exposure at *T_0_* and *T_1_*) that we observed in the main analysis.


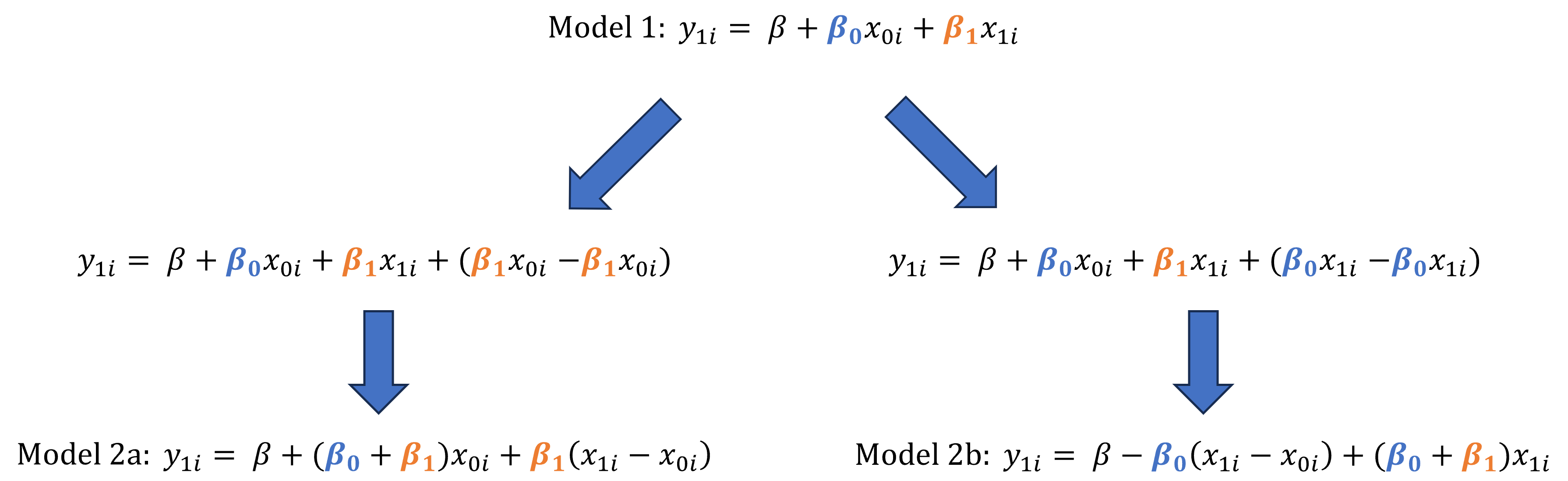


Note: $y_{1i}$ indicates the score of externalizing problems for the person $i$; $\beta$ indicates the intercept; $x_{0i}$ and $x_{1i}$ indicate the green space exposure at *T_0_* (i.e., prenatal) and *T_1_* for person $i$, respectively; $x_{1i}-x_{0i}$ indicates the green space exposure changes from *T_0_* to *T_1_* for the person $i$; $\beta_{0}$ and $\beta_{1}$indicate the effect coefficients. Terms for covariates and the residuals were removed from the equations for simplicity.

# **Table S1.** Results of the attrition analysis.

|  | *N* (%) or mean ± standard deviation | | | |
| --- | --- | --- | --- | --- |
| Variables | Total Sample  (*N* = 2,229) | Excluded  (*N* = 753) | Included  (*N* = 1,476) | *P-value* |
| *Outcomes* |  |  |  |  |
| Externalizing problems | 0.27 ± 0.20 | 0.28 ± 0.20 | 0.27 ± 0.19 | 0.433 |
| Missing | 42 | 18 | 24 |  |
| Internalizing problems | 0.36 ± 0.24 | 0.36 ± 0.25 | 0.36 ± 0.23 | 0.763 |
| Missing | 59 | 25 | 34 |  |
| Tobacco use |  |  |  |  |
| Never | 1,898 (86.3%) | 610 (82.5%) | 1,288 (88.2%) | <0.001 |
| At least once | 302 (13.7%) | 129 (17.5%) | 173 (11.8%) |  |
| Missing | 29 | 14 | 15 |  |
| Alcohol use |  |  |  |  |
| Never | 1,518 (69.1%) | 507 (68.6%) | 1,011 (69.3%) | 0.512 |
| At least once | 680 (31.9%) | 232 (31.4%) | 448 (30.7%) |  |
| Missing | 31 | 14 | 17 |  |
| *Mediators* |  |  |  |  |
| Gestational age | 39.76 ± 2.04 | 39.52 ± 2.39 | 39.87 ± 1.85 | 0.012 |
| Missing | 61 | 51 | 10 |  |
| Birthweight | 6.78 ± 1.23 | 6.66 ± 1.32 | 6.84 ± 1.19 | 0.018 |
| Missing | 96 | 72 | 24 |  |
| *Demographics* |  |  |  |  |
| Age in *T_1_* | 11.1 ± 0.6 | 11.1 ± 0.6 | 11.1 ± 0.5 | 0.098 |
| Sex at birth |  |  |  |  |
| Female | 1,131 (50.7%) | 372 (49.4%) | 759 (51.4%) | 0.391 |
| Male | 1,098 (49.3%) | 381 (50.6%) | 717 (48.6%) |  |
| Ethnicity |  |  |  |  |
| Native | 1,992 (89.4%) | 609 (80.9%) | 1,383 (93.7%) | <0.001 |
| Immigrant | 237 (10.6%) | 144 (19.1%) | 93 (6.3%) |  |
| Family socioeconomic status (*T_1_*) | -0.05 ± 0.80 | -0.26 ± 0.81 | 0.05 ± 0.77 | 0.003 |
| Missing | 42 | 34 | 8 |  |
| Parental divorce before *T_1_* |  |  |  |  |
| Not divorced | 1,752 (78.6%) | 497 (66.0%) | 1,255 (85.0%) | <0.001 |
| Divorced | 477 (21.4%) | 256 (34.0%) | 221 (15.0%) |  |
| Parental externalizing problems (*T_1_*) | 0.14 ± 0.42 | 0.22 ± 0.52 | 0.11 ± 0.36 | <0.001 |
| Missing | 65 | 52 | 13 |  |
| Parental internalizing problems (*T_1_*) | 0.55 ± 0.79 | 0.64 ± 0.84 | 0.51 ± 0.77 | <0.001 |
| Missing | 71 | 55 | 16 |  |
| Prenatal and perinatal complications |  |  |  |  |
| No complications | 869 (40.1%) | 268 (38.2%) | 601 (41.0%) | 0.454 |
| 1-4 complications | 968 (44.7%) | 321 (45.8%) | 647 (44.1%) |  |
| >5 complications | 330 (15.2%) | 112 (16.0%) | 218 (14.9%) |  |
| Missing | 62 | 52 | 10 |  |
| Maternal age | 29.3 ± 4.6 | 27.9 ± 4.8 | 30.0 ± 4.3 | <0.001 |
| Missing | 114 | 81 | 33 |  |
| Maternal tobacco use |  |  |  |  |
| Never | 1,508 (69.5%) | 460 (65.6%) | 1,048 (71.4%) | 0.003 |
| 1-10 cigarettes/day | 512 (23.6%) | 177 (25.2%) | 335 (22.8%) |  |
| >10 cigarettes/day | 148 (6.9%) | 64 (9.1%) | 84 (5.7%) |  |
| Missing | 61 | 52 | 9 |  |
| Maternal alcohol use |  |  |  |  |
| Never | 1,761 (81.3%) | 600 (85.8%) | 1,161 (79.2%) | <0.001 |
| 1-3 glasses/week | 379 (17.5%) | 92 (13.2%) | 287 (19.6%) |  |
| >3 glasses/week | 25 (1.2%) | 7 (1.0%) | 18 (1.2%) |  |
| Missing | 64 | 54 | 10 |  |

Note: *P*-*values* estimated using chi-squared tests for categorical variables and Kruskal-Wallis tests for continuous variables were used to indicate the differences between the included and excluded samples.

# **Table S2.** Bivariate correlations for the study variables.

|  | 1 | 2 | 3 | 4 | 5 | 6 | 7 | 8 | 9 | 10 | 11 | 12 | 13 | 14 | 15 | 16 | 17 | 18 | 19 | 20 | 21 | 22 |
| --- | --- | --- | --- | --- | --- | --- | --- | --- | --- | --- | --- | --- | --- | --- | --- | --- | --- | --- | --- | --- | --- | --- |
| Main variables |  |  |  |  |  |  |  |  |  |  |  |  |  |  |  |  |  |  |  |  |  |  |
| 1.Externalizing problems | -- |  |  |  |  |  |  |  |  |  |  |  |  |  |  |  |  |  |  |  |  |  |
| 2.Internalizing problems | .53^**^ | -- |  |  |  |  |  |  |  |  |  |  |  |  |  |  |  |  |  |  |  |  |
| 3.Tobacco use | .27^**^ | .16^**^ | -- |  |  |  |  |  |  |  |  |  |  |  |  |  |  |  |  |  |  |  |
| 4.Alcohol use | .33^**^ | .13^**^ | .33^**^ | -- |  |  |  |  |  |  |  |  |  |  |  |  |  |  |  |  |  |  |
| 5.Gestational age | .02 | .00 | .05^*^ | .05 | -- |  |  |  |  |  |  |  |  |  |  |  |  |  |  |  |  |  |
| 6.Birthweight | .07^**^ | -.04 | .03 | .05 | .34^**^ | -- |  |  |  |  |  |  |  |  |  |  |  |  |  |  |  |  |
| 7.Green space (*T_0_*) | .05 | .06^*^ | .01 | .03 | -.02 | .00 | -- |  |  |  |  |  |  |  |  |  |  |  |  |  |  |  |
| 8.Green space (*T_1_*) | -.01 | .03 | .00 | -.03 | -.03 | -.01 | .67^**^ | -- |  |  |  |  |  |  |  |  |  |  |  |  |  |  |
| Covariates |  |  |  |  |  |  |  |  |  |  |  |  |  |  |  |  |  |  |  |  |  |  |
| 9.Age in *T_1_* | .05^*^ | -.08^**^ | .03 | .12^**^ | .05 | .03 | -.27^**^ | -.32^**^ | -- |  |  |  |  |  |  |  |  |  |  |  |  |  |
| 10.Sex at birth (*ref: female*) | .18^**^ | -.12^**^ | .06^*^ | .17^**^ | -.02 | .15^**^ | .01 | -.01 | .00 | -- |  |  |  |  |  |  |  |  |  |  |  |  |
| 11.Ethnicity (*ref: native*) | -.01 | .03 | .05^*^ | -.04 | -.06^*^ | -.09^**^ | -.11^**^ | -.10^**^ | .09^**^ | -.02 | -- |  |  |  |  |  |  |  |  |  |  |  |
| 12.Family socioeconomic status (*T_1_*) | -.02 | -.06^*^ | -.11^**^ | -.01 | -.01 | .07^*^ | -.12^**^ | -.09^**^ | .03 | .02 | -.16^**^ | -- |  |  |  |  |  |  |  |  |  |  |
| 13.Parental divorce before *T_1_* | .04 | .03 | .11^**^ | -.01 | -.01 | -.03 | -.04 | -.07^*^ | .06^*^ | -.05 | .04 | -.17^**^ | -- |  |  |  |  |  |  |  |  |  |
| 14.Parental externalizing problems (*T_1_*) | .02 | .01 | .11^**^ | .03 | .02 | .01 | -.03 | -.07^**^ | .04 | .01 | .01 | -.12^**^ | .34^**^ | -- |  |  |  |  |  |  |  |  |
| 15.Parental internalizing problems (*T_1_*) | .03 | .03 | .06^*^ | -.02 | .04 | .03 | .03 | .00 | .01 | -.01 | .03 | -.05^*^ | .16^**^ | .29^**^ | -- |  |  |  |  |  |  |  |
| 16.Prenatal and perinatal complications | -.04 | -.01 | .01 | -.03 | -.03 | -.02 | -.03 | -.03 | .04 | .04 | -.01 | .02 | .05^*^ | .11^**^ | .17^**^ | -- |  |  |  |  |  |  |
| 17.Maternal age | -.04 | -.05 | -.06^*^ | -.02 | -.03 | .07^**^ | -.11^**^ | -.11^**^ | .03 | .00 | .00 | .33^**^ | -.03 | .00 | .04 | .03 | -- |  |  |  |  |  |
| 18.Maternal tobacco use | .05 | .00 | .12^**^ | .06^*^ | .02 | -.14^**^ | .01 | .01 | .00 | -.04 | -.01 | -.19^**^ | .09^**^ | .19^**^ | .06^*^ | .00 | -.08^**^ | -- |  |  |  |  |
| 19.Maternal alcohol use | .02 | -.01 | -.02 | -.02 | .01 | .03 | -.10^**^ | -.09^**^ | .06^*^ | .01 | -.04 | .21^**^ | .03 | .10^**^ | .03 | .00 | .22^**^ | .09^**^ | -- |  |  |  |
| 20.Deprivation (prenatal) | -.01 | -.04 | .02 | .00 | .05 | .02 | -.28^**^ | -.11^**^ | .12^**^ | .00 | .14^**^ | -.19^**^ | .11^**^ | .10^**^ | .01 | -.03 | -.06^*^ | .07^**^ | .02 | -- |  |  |
| 21.Social fragmentation (prenatal) | -.03 | -.08^**^ | .00 | -.01 | .03 | .01 | -.72^**^ | -.48^**^ | .30^**^ | .01 | .10^**^ | .08^**^ | .11^**^ | .08^**^ | .01 | .02 | .10^**^ | .06^*^ | .13^**^ | .57^**^ | -- |  |
| 22.Deprivation (*T_1_*) | .02 | .02 | .06^*^ | .02 | .01 | .00 | -.09^**^ | -.15^**^ | .10^**^ | .00 | .17^**^ | -.27^**^ | .16^**^ | .12^**^ | .07^*^ | -.04 | -.05 | .08^**^ | -.02 | .60^**^ | .31^**^ | -- |
| 23.Social fragmentation (*T_1_*) | -.01 | -.03 | .02 | .04 | .04 | -.01 | -.47^**^ | -.58^**^ | .32^**^ | .02 | .15^**^ | .01 | .13^**^ | .12^**^ | .04 | .02 | .10^**^ | .05 | .09^**^ | .40^**^ | .69^**^ | .60^**^ |

Note: **. Correlation is significant at the 0.01 level (*P* value); *. Correlation is significant at the 0.05 level (*P* value).

# **Table S3.** Results of the main analysis.

|  | Gestational age | Birth weight | Externalizing problems | Internalizing problems | Tobacco use | Alcohol use | Green space (*T_1_*) |
| --- | --- | --- | --- | --- | --- | --- | --- |
| *Mediators* |  |  |  |  |  |  |  |
| Gestational age | - | - | -0.015 (-0.073, 0.044) | -0.032 (-0.085, 0.020) | 0.071 (-0.019, 0.162) | 0.033 (-0.036, 0.103) | - |
| Birthweight | - | - | 0.053 (-0.005, 0.111) | 0.009 (-0.048, 0.066) | 0.054 (-0.044, 0.152) | 0.017 (-0.057, 0.092) | - |
| *Exposures* |  |  |  |  |  |  |  |
| Green space (*T_0_*) |  |  |  |  |  |  |  |
| Direct effect | 0.015 (-0.068, 0.097) | 0.002 (-0.078, 0.082) | 0.119 (0.028, 0.210) | 0.019 (-0.073, 0.112) | 0.046 (-0.106, 0.197) | 0.042 (-0.081, 0.165) | 0.690 (0.639, 0.742) |
| Total indirect effects | - | - | -0.078 (-0.135, -0.022) | -0.057 (-0.117, 0.004) | 0.000 (-0.097, 0.097) | -0.014 (-0.092, 0.065) | - |
| Via gestational age | - | - | 0.000 (-0.002, 0.001) | 0.000 (-0.003, 0.002) | 0.001 (-0.005, 0.007) | 0.000 (-0.002, 0.003) | - |
| Via birthweight | - | - | 0.000 (-0.004, 0.004) | 0.000 (-0.001, 0.001) | 0.000 (-0.004, 0.004) | 0.000 (-0.001, 0.002) | - |
| Via green space(*T_1_*) | - | - | -0.078 (-0.135, -0.022) | -0.056 (-0.116, 0.004) | -0.001 (-0.098, 0.096) | -0.014 (-0.093, 0.064) | - |
| Total effect | - | - | 0.040 (-0.036, 0.116) | -0.037 (-0.112, 0.037) | 0.046 (-0.078, 0.169) | 0.028 (-0.068, 0.125) | - |
| Green space (*T_1_*) | - | - | -0.113 (-0.195, -0.032) | -0.081 (-0.168, 0.005) | -0.002 (-0.142, 0.138) | -0.021 (-0.134, 0.093) | - |
| Covariances |  |  |  |  |  |  |  |
| Gestational age | - | - | - | - | - | - | - |
| Birthweight | 0.438 (0.403, 0.472) | - | - | - | - | - | - |
| Externalizing problems | - | - | - | - | - | - | - |
| Internalizing problems | - | - | 0.553 (0.524, 0.583) | - | - | - | - |
| Tobacco use | - | - | 0.369 (0.298,0.440) | 0.193 (0.113, 0.273) | - |  | - |
| Alcohol use | - | - | 0.368 (0.309, 0.426) | 0.181 (0.118, 0.244) | 0.552 (0.470, 0.635) | - | - |

Note: All coefficients were standardized (*β_STDYX_*), interpreted as ‘the SD unit difference in the outcome/mediator per SD unit difference in the mediator/exposure’. For binary outcomes (tobacco and alcohol use), the SD of the continuous latent response variable was used for standardization. Gestational age is measured in weeks; birthweight is measured in pounds.

# **Table S4.** Results of the sensitivity analyses to adjusting for urbanicity, PM_2.5_, or traffic noise

|  | Externalizing problems | Internalizing problems | Tobacco use | Alcohol use |
| --- | --- | --- | --- | --- |
| Main model |  |  |  |  |
| Green space (*T_0_*) | 0.119 (0.028, 0.210) | 0.019 (-0.073, 0.112) | 0.046 (-0.106, 0.197) | 0.042 (-0.081, 0.165) |
| Green space (*T_1_*) | -0.113 (-0.195, -0.032) | -0.081 (-0.168, 0.005) | -0.002 (-0.142, 0.138) | -0.021 (-0.134, 0.093) |
| Main model + urbanicity |  |  |  |  |
| Green space (*T_0_*) | 0.096 (-0.003, 0.195) | 0.030 (-0.073, 0.132) | 0.004 (-0.166, 0.173) | 0.043 (-0.094, 0.179) |
| Green space (*T_1_*) | -0.116 (-0.202, -0.031) | -0.068 (-0.161, 0.026) | -0.032 (-0.185, 0.120) | 0.011 (-0.113, 0.135) |
| Main + PM_2.5_ |  |  |  |  |
| Green space (*T_0_*) | 0.156 (-0.102, 0.413) | 0.044 (-0.129, 0.217) | 0.007 (-0.205, 0.220) | 0.045 (-0.132, 0.221) |
| Green space (*T_1_*) | -0.112 (-0.407, 0.184) | -0.082 (-0.261, 0.096) | 0.052 (-0.176, 0.280) | -0.023 (-0.218, 0.173) |
| Main + noise |  |  |  |  |
| Green space (*T_0_*) | 0.123 (0.030, 0.216) | 0.015 (-0.076, 0.107) | 0.047 (-0.111, 0.205) | 0.037 (-0.086, 0.160) |
| Green space (*T_1_*) | -0.103 (-0.189, -0.017) | -0.063 (-0.157, 0.030) | -0.023 (-0.174, 0.129) | -0.009 (-0.131, 0.113) |

Note: All coefficients were standardized (*β_STDYX_*), interpreted as ‘the SD unit difference in the outcome/mediator per SD unit difference in the mediator/exposure’. For binary outcomes (tobacco and alcohol use), the SD of the continuous latent response variable was used for standardization. Given the null mediation of gestational age and birthweight observed in the main analysis, we excluded these two variables from models controlling for urbanicity, PM_2.5_, and noise to facilitate model convergence and comparison between the effect sizes for green space at *T_0_* and *T_1_* (both are total effects).

# **Table S5.** Results of the model using prenatal green space exposure assessed with seasonal differences in NDVI during pregnancy incorporated.

|  | Gestational age | Birth weight | Externalizing problems | Internalizing problems | Tobacco use | Alcohol use | Green space (*T_1_*) |
| --- | --- | --- | --- | --- | --- | --- | --- |
| Mediators |  |  |  |  |  |  |  |
| Gestational age | - | - | -0.015 (-0.073, 0.042) | -0.029 (-0.081, 0.024) | 0.075 (-0.015, 0.165) | 0.030 (-0.039, 0.099) | - |
| Birthweight | - | - | 0.056 (-0.002, 0.114) | 0.008 (-0.050, 0.066) | 0.055 (-0.042, 0.152) | 0.020 (-0.055, 0.095) | - |
| Exposures |  |  |  |  |  |  |  |
| Green space (*T_0_*) |  |  |  |  |  |  |  |
| Direct effect | 0.022 (-0.051, 0.095) | -0.010 (-0.084, 0.064) | 0.102 (0.017, 0.187) | -0.004 (-0.090, 0.083) | 0.076 (-0.066, 0.218) | 0.009 (-0.110, 0.128) | 0.649 (0.602, 0.696) |
| Total indirect effects | - | - | -0.070 (-0.123, -0.018) | -0.043 (-0.100, 0.015) | -0.014 (-0.105, 0.076) | -0.006 (-0.082, 0.069) | - |
| Via gestational age | - | - | 0.000 (-0.002, 0.001) | -0.001 (-0.003, 0.002) | 0.002 (-0.004, 0.008) | 0.001 (-0.002, 0.003) | - |
| Via birthweight | - | - | -0.001 (-0.005, 0.004) | 0.000 (-0.001, 0.001) | -0.001 (-0.005, 0.004) | 0.000 (-0.002, 0.002) | - |
| Via green space(*T_1_*) | - | - | -0.070 (-0.122, -0.017) | -0.042 (-0.099, 0.016) | -0.015 (-0.105, 0.075) | -0.007 (-0.082, 0.069) | - |
| Total effect | - | - | 0.031 (-0.039, 0.102) | -0.046 (-0.115, 0.023) | 0.062 (-0.055, 0.178) | 0.003 (-0.089, 0.095) | - |
| Green space (*T_1_*) | - | - | -0.107 (-0.188, -0.027) | -0.065 (-0.153, 0.024) | -0.024 (-0.162, 0.115) | -0.010 (-0.127, 0.106) | - |
| Covariances |  |  |  |  |  |  |  |
| Gestational age | - | - | - | - | - | - | - |
| Birthweight | 0.439 (0.405, 0.473) | - | - | - | - | - | - |
| Externalizing problems | - | - | - | - | - | - | - |
| Internalizing problems | - | - | 0.553 (0.522, 0.583) | - | - | - | - |
| Tobacco use | - | - | 0.365 (0.294,0.436) | 0.187 (0.107, 0.268) | - |  | - |
| Alcohol use | - | - | 0.366 (0.308, 0.425) | 0.176 (0.113, 0.240) | 0.549 (0.467, 0.632) | - | - |

Note: All coefficients were standardized (*β_STDYX_*), interpreted as ‘the SD unit difference in the outcome/mediator per SD unit difference in the mediator/exposure’. For binary outcomes (tobacco and alcohol use), the SD of the continuous latent response variable was used for standardization. Gestational age is measured in weeks; birth weight is measured in pounds.

# **Table S6.** Results of the complete case (*n*=1,382) analysis.

|  | Gestational age | Birth weight | Externalizing problems | Internalizing problems | Tobacco use | Alcohol use | Green space (*T_1_*) |
| --- | --- | --- | --- | --- | --- | --- | --- |
| Mediators |  |  |  |  |  |  |  |
| Gestational age | - | - | -0.014 (-0.073, 0.046) | -0.033 (-0.088, 0.021) | 0.079 (-0.014, 0.172) | 0.030 (-0.040, 0.100) | - |
| Birthweight | - | - | 0.050 (-0.009, 0.109) | 0.009 (-0.048, 0.067) | 0.040 (-0.060, 0.140) | 0.018 (-0.058, 0.093) | - |
| Exposures |  |  |  |  |  |  |  |
| Green space (*T_0_*) |  |  |  |  |  |  |  |
| Direct effect | 0.009 (-0.076, 0.094) | 0.000 (-0.082, 0.082) | 0.114 (0.023, 0.205) | 0.005 (-0.087, 0.098) | 0.065 (-0.092, 0.223) | 0.054 (-0.072, 0.180) | 0.683 (0.631, 0.736) |
| Total indirect effects | - | - | -0.072 (-0.128, -0.016) | -0.048 (-0.107, 0.011) | -0.006 (-0.105, 0.093) | -0.021 (-0.101, 0.058) | - |
| Via gestational age | - | - | 0.000 (-0.001, 0.001) | 0.000 (-0.003, 0.003) | 0.001 (-0.006, 0.007) | 0.000 (-0.002, 0.003) | - |
| Via birthweight | - | - | 0.000 (-0.004, 0.004) | 0.000 (-0.001, 0.001) | 0.000 (-0.003, 0.003) | 0.000 (-0.001, 0.001) | - |
| Via green space(*T_1_*) | - | - | -0.072 (-0.128, -0.017) | -0.048 (-0.107, 0.012) | -0.007 (-0.105, 0.092) | -0.022 (-0.101, 0.058) | - |
| Total effect | - | - | 0.042 (-0.035, 0.119) | -0.042 (-0.119, 0.034) | 0.060 (-0.069, 0.188) | 0.032 (-0.067, 0.132) | - |
| Green space (*T_1_*) | - | - | -0.106 (-0.187, -0.024) | -0.070 (-0.156, 0.017) | -0.010 (-0.154, 0.134) | -0.032 (-0.148, 0.084) | - |
| Covariances |  |  |  |  |  |  |  |
| Gestational age | - | - | - | - | - | - | - |
| Birthweight | 0.448 (0.414, 0.483) | - | - | - | - | - | - |
| Externalizing problems | - | - | - | - | - | - | - |
| Internalizing problems | - | - | 0.549 (0.519, 0.579) | - | - | - | - |
| Tobacco use | - | - | 0.384 (0.313, 0.456) | 0.198 (0.117, 0.279) | - |  | - |
| Alcohol use | - | - | 0.373 (0.314, 0.433) | 0.188 (0.124, 0.252) | 0.552 (0.467, 0.637) | - | - |

Note: All coefficients were standardized (*β_STDYX_*), interpreted as ‘the SD unit difference in the outcome/mediator per SD unit difference in the mediator/exposure’. For binary outcomes (tobacco and alcohol use), the SD of the continuous latent response variable was used for standardization. Gestational age is measured in weeks; birth weight is measured in pounds.

**
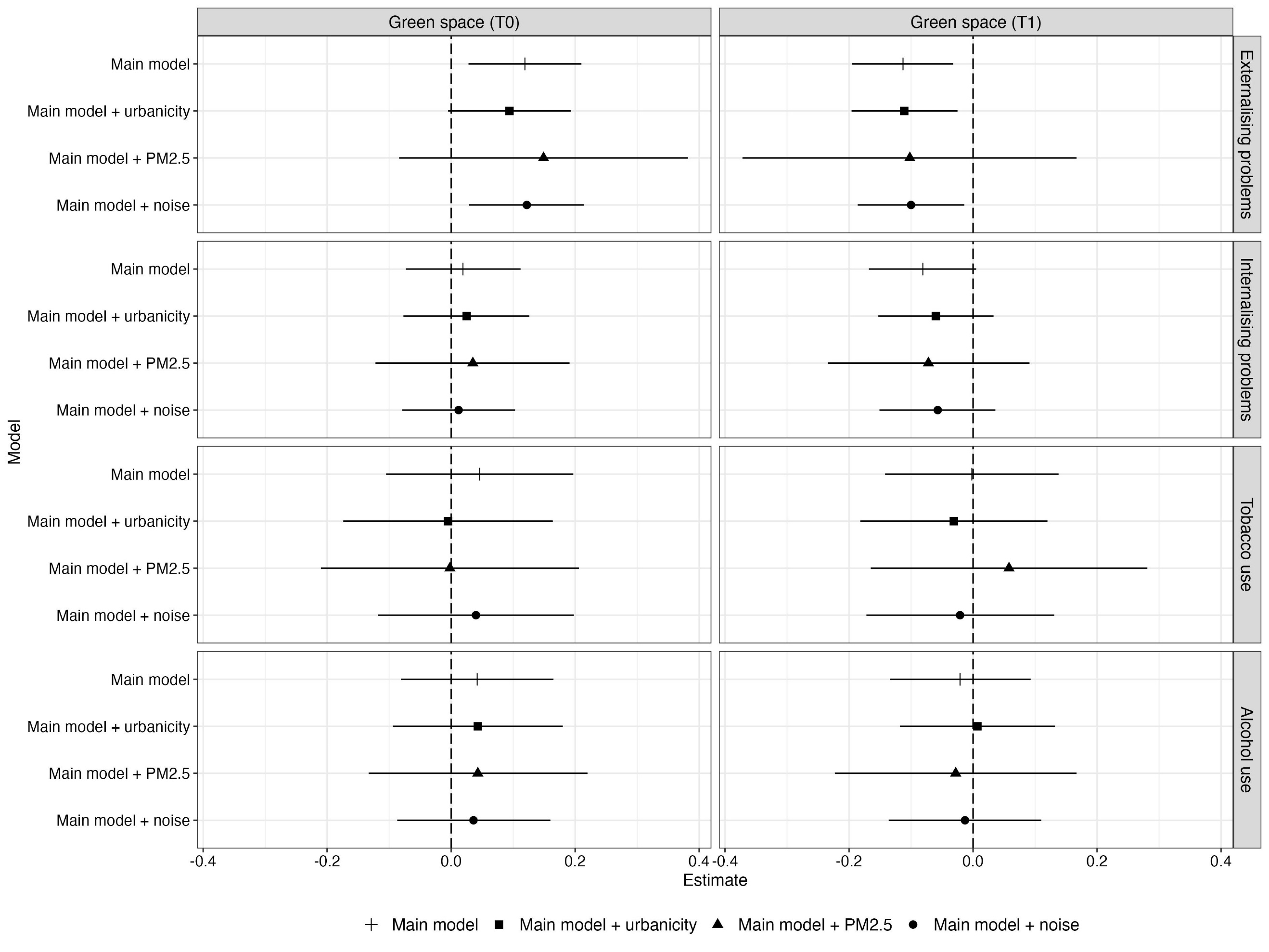
**

**Figure S1.** Associations of green space at *T_0_* and *T_1_* with externalizing problems, internalizing problems, tobacco use, and alcohol use from models with different area-level factors included. Green space at *T_0_* and *T_1_* were included in the same model. Given the null mediation of gestational age and birthweight observed in the main analysis, we excluded these two variables from models controlling for urbanicity, PM_2.5_, and noise to facilitate model convergence and comparison between the effect sizes for green space at *T_0_* and *T_1_* (both are total effects). All coefficients were standardized (*β_STDYX_*), interpreted as ‘the SD unit difference in the outcome/mediator per SD unit difference in the mediator/exposure’. For binary outcomes (tobacco and alcohol use), the SD of the continuous latent response variable was used for standardization.

**
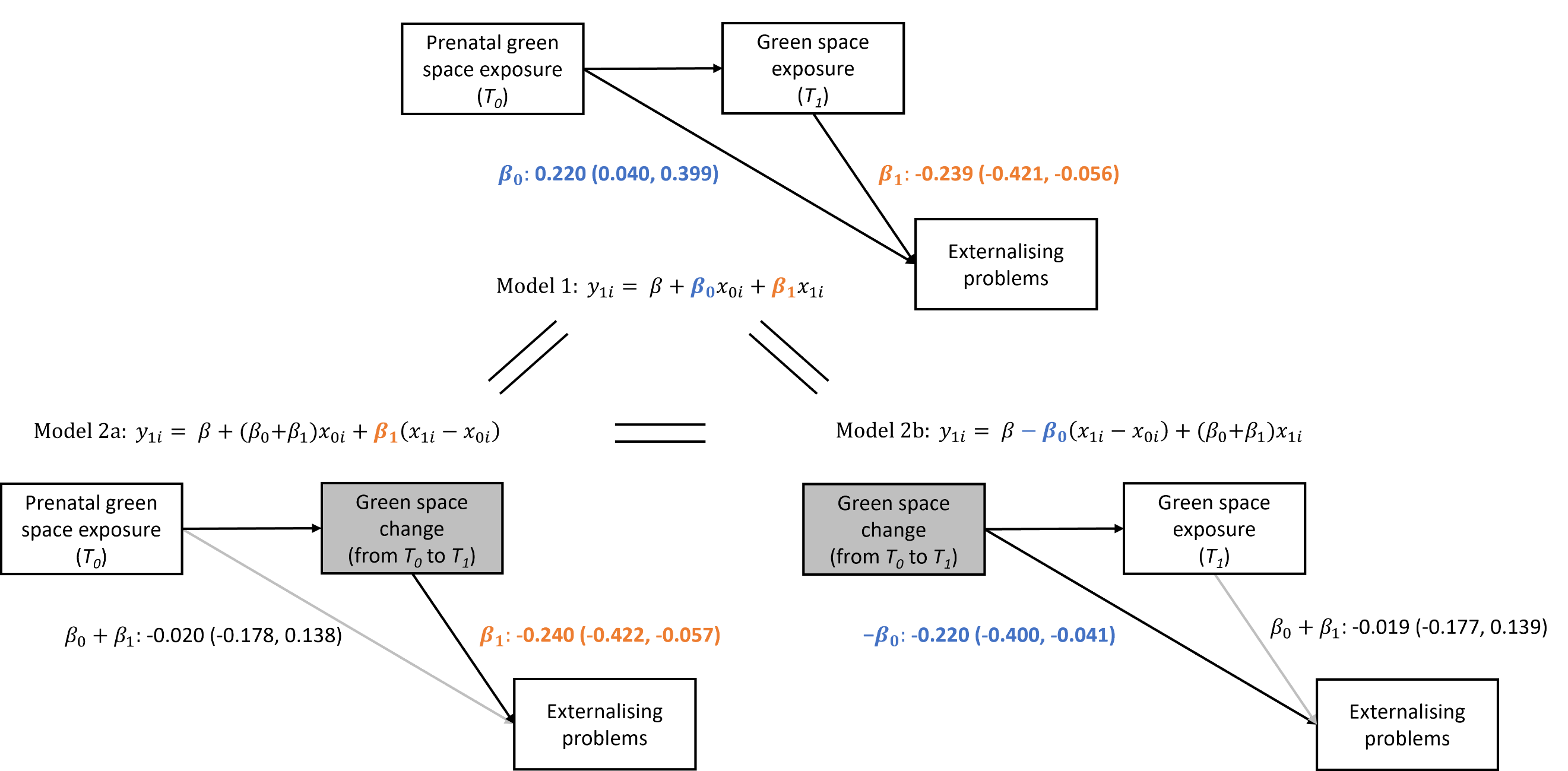
**

# **Figure S2.** Unstandardized results of the post-hoc analysis. All covariates were controlled as the model in the main analysis. Gestational age and birthweight (the assumed mediators) were removed for simplicity.

**References**

1. Vollebergh WAM, Ten Have M, Dekovic M, et al. Mental health in immigrant children in the Netherlands. *Social Psychiatry and Psychiatric Epidemiology*. 2005;40(6):489-496. doi:10.1007/s00127-005-0906-1

2. Ganzeboom HB, Treiman DJ. Internationally comparable measures of occupational status for the 1988 International Standard Classification of Occupations. *Social science research*. 1996;25(3):201-239.

3. Ormel J, Oldehinkel AJ, Ferdinand RF, et al. Internalizing and externalizing problems in adolescence: general and dimension-specific effects of familial loadings and preadolescent temperament traits. *Psychological Medicine*. 2005;35(12):1825-1835. doi:10.1017/s0033291705005829

4. Buschgens CJM, Swinkels SHN, van Aken MAG, Ormel J, Verhulst FC, Buitelaar JK. Externalizing behaviors in preadolescents: familial risk to externalizing behaviors, prenatal and perinatal risks, and their interactions. *European Child & Adolescent Psychiatry*. 2009/02/01 2009;18(2):65-74. doi:10.1007/s00787-008-0704-x

5. Marceau K, Brick LA, Pasman JA, Knopik VS, Reijneveld SA. Interactions between Genetic, Prenatal, Cortisol, and Parenting Influences on Adolescent Substance Use and Frequency: A TRAILS Study. *European Addiction Research*. 2021;28(3):176-185. doi:10.1159/000519864

6. Hagedoorn P, Groenewegen PP, Roberts H, Helbich M. Is suicide mortality associated with neighbourhood social fragmentation and deprivation? A Dutch register-based case-control study using individualised neighbourhoods. *Journal of Epidemiology and Community Health*. 2020;74(2):197. doi:10.1136/jech-2019-212699

7. Van Donkelaar A, Martin RV, Li C, Burnett RT. Regional Estimates of Chemical Composition of Fine Particulate Matter Using a Combined Geoscience-Statistical Method with Information from Satellites, Models, and Monitors. *Environmental Science & Technology*. 2019;53(5):2595-2611. doi:10.1021/acs.est.8b06392

8. PBL. Road-, Rail- and Air-traffic Noise in the Netherlands in 2000-2008. Accessed March 6, 2022, https://www.gecco.nl/app/download/7852786664/Metadatasheet%20-%20Noise%20Road-%20Rail-%20Air%20Traffic.pdf?t=1578389028

9. VanderWeele TJ, Ding P. Sensitivity Analysis in Observational Research: Introducing the E-Value. *Annals of Internal Medicine*. 2017/08/15 2017;167(4):268-274. doi:10.7326/M16-2607

10. Maitre L, Julvez J, López-Vicente M, et al. Early-life environmental exposure determinants of child behavior in Europe: A longitudinal, population-based study. *Environment International*. 2021/08/01/ 2021;153:106523. doi:10.1016/j.envint.2021.106523

11. Weuve J, Tchetgen Tchetgen EJ, Glymour MM, et al. Accounting for Bias Due to Selective Attrition: The Example of Smoking and Cognitive Decline. *Epidemiology*. 2012;23(1)

12. Cole SR, Stuart EA. Generalizing Evidence From Randomized Clinical Trials to Target Populations: The ACTG 320 Trial. *American Journal of Epidemiology*. 2010;172(1):107-115. doi:10.1093/aje/kwq084

13. Hernán MÁ, Brumback B, Robins JM. Marginal Structural Models to Estimate the Causal Effect of Zidovudine on the Survival of HIV-Positive Men. *Epidemiology*. 2000;11(5)

14. Cole SR, Hernán MA, Margolick JB, Cohen MH, Robins JM. Marginal Structural Models for Estimating the Effect of Highly Active Antiretroviral Therapy Initiation on CD4 Cell Count. *American Journal of Epidemiology*. 2005;162(5):471-478. doi:10.1093/aje/kwi216

15. Haneuse S, Schildcrout J, Crane P, Sonnen J, Breitner J, Larson E. Adjustment for Selection Bias in Observational Studies with Application to the Analysis of Autopsy Data. *Neuroepidemiology*. 2009;32(3):229-239. doi:10.1159/000197389

16. Cole SR, Hernán MA. Constructing Inverse Probability Weights for Marginal Structural Models. *American Journal of Epidemiology*. 2008;168(6):656-664. doi:10.1093/aje/kwn164
