# Supplementary material for: Prenatal exposure to green space and mental health in early adolescence: findings from the TRAILS study
Source: Am J Epidemiol. 2024 Sep 24;194(7):1949–58. doi: 10.1093/aje/kwae373 (PMC12234223; doi:10.1093/aje/kwae373)
Supplement: Web_Material_kwae373 [file web_material_kwae373.zip › title page of supplementary data.docx]

**Supplemental Material**

**Prenatal exposure to green space and mental health in early adolescence: Findings from the TRAILS study**

Yi Zeng, Gonneke W. J. M. Stevens, Tomáš Paus, & Marco Helbich

**Table of contents**

**Appendix S1.** In-depth information on some covariates.

**Appendix S2.** The sensitivity analysis for evaluating the potential for unmeasured confounding.

**Appendix S3.** The sensitivity analysis for evaluating and correcting sample selection bias.

**Appendix S4.** The assessment of prenatal green space exposure considering seasonal differences in NDVI.

**Appendix S5.** The model conversion.

**Table S1.** Results of the attrition analysis.

**Table S2.** Bivariate correlations for the study variables.

**Table S3.** Results of the main analysis.

**Table S4.** Results of the sensitivity analyses to adjusting for urbanicity, PM_2.5_, or traffic noise.

**Table S5.** Results of the model using prenatal green space exposure assessed with seasonal differences in NDVI during pregnancy incorporated.

**Table S6.** Results of the complete case analysis.

**Figure S1.** Associations of green space at *T_0_* and *T_1_* with externalizing problems, internalizing problems, tobacco use, and alcohol use from models with different area-level factors included.

**Figure S2.** Unstandardized results of the post-hoc analysis.
